# Exploring pathways leading to stillbirths and gaps in postnatal care among affected women in a rural north Indian district: A qualitative study using the social autopsy lens

**DOI:** 10.1371/journal.pone.0347994

**Published:** 2026-05-13

**Authors:** Barsha Gadapani Pathak, Sonia Maurya, Shruti Bisht, Pranay Vats, Reema Mukerjee, Vinod Kumar Anand, Sarmila Mazumder

**Affiliations:** 1 Society for Applied Studies, New Delhi, India; 2 Centre for Intervention Science in Maternal and Child Health, Centre for International Health, Department of Global Public Health and Primary Care, University of Bergen, Bergen, Norway; 3 Division of Reproductive, Child Health and Nutrition, Indian Council of Medical Research, New Delhi, India; Kalahandi University, INDIA

## Abstract

**Background:**

Globally, stillbirth is a silent yet significant contributor to perinatal mortality. India has several national maternal and child health programs, yet, the stillbirths remain poorly reviewed, subject to suboptimal surveillance, are under-reported, and often absent from many programmatic priorities. This study aimed to explore the pathways, delays leading to stillbirths and continuum of care in postpartum phase among affected women in a rural district of North India using a social autopsy tool.

**Methods:**

This qualitative study was a component of an ongoing implementation research to reduce stillbirth in Palwal district of Haryana. In-depth interviews were conducted with 25 women who had experienced stillbirths using a specially designed social autopsy tool. Additionally, five healthcare providers were interviewed for getting a holistic insight on pathway leading to stillbirth. Thematic analysis was performed using NVivo 16 software, and both deductive (Three Delays Model framework) and inductive approaches were applied to identify delays and contextual factors influencing care-seeking, and postpartum experiences.

**Results:**

The pathways identified as contributor stillbirths were: (i) low utilization of antenatal and delivery care, driven by cultural beliefs, low perceived risk, and gendered power dynamics; (ii) delays in accessing care due to poor transport, restrictive social norms, and infrastructural gaps; (iii) poor quality of care, characterized by disrespectful treatment, inappropriate referrals, and inadequate intrapartum management. Additionally, a complete absence of postpartum follow-up, grief support, or mental health care was found, reflecting a neglected dimension of postpartum maternal care following stillbirths.

**Conclusions:**

Stillbirths in this setting are the consequence of interlinked socio-cultural, health system, and gendered vulnerabilities. The implications of stillbirths extended beyond immediate consequences to include enduring mental health and social impacts, ultimately undermining women’s confidence, well-being and preparedness for future pregnancies. There is an urgent need to integrate stillbirth reporting, respectful maternity care, and post-loss psychosocial support within India’s maternal health programs.

**Trial Registration:**

The primary implementation research was registered prospectively in the Clinical Trial Registry of India (CTRI): CTRI/2024/07/069796 [**Registered on: 02/07/2024**].

## Background

Stillbirth remains a critical but under-addressed public health issue, with nearly two million cases reported annually worldwide. The burden is disproportionately higher in low- and middle-income countries (LMICs), particularly in sub-Saharan Africa and South Asia, where 98% of all stillbirths occur, with rates reaching as high as 39.1 per 1,000 births. [1] Among these, India carries the highest burden, contributing to nearly two-thirds of global stillbirths, underscoring the urgent need for targeted interventions to prevent these losses. [2]

Despite its high burden and devastating consequences, stillbirth remains largely invisible in health agendas, often overshadowed by neonatal and maternal mortality. Beyond the medical complications, the psychosocial and emotional impact of stillbirth on mothers and families is profound, leading to stigma, grief, depression, and social isolation.[3,4] On the provider side, stillbirths are frequently misclassified, underreported, or dismissed as inevitable, contributing to data invisibility and limiting programmatic response.[5] In some cases, blame is implicitly or explicitly shifted to the women, reinforcing feelings of guilt and marginalization. These challenges are compounded by gaps in data, poor record-keeping, inconsistent use of standard case definitions, and the absence of comprehensive reviews or accountability mechanisms. [5] Consequently, there is a persistent underestimation of the burden, and a lack of actionable data that hinders the identification of modifiable contributory factors and delays the development of effective interventions. [5]

Stillbirths result from a complex interplay of medical, social, and systemic determinants. Maternal infections (syphilis, malaria, HIV), obstetric complications (uncontrolled diabetes, hypertensive disorders, placental abnormalities), and fetal conditions (prematurity, low birth weight, congenital anomalies) significantly elevate the risk. Additionally, women who experience one stillbirth face a higher risk of recurrence in subsequent pregnancies. Notably, up to 50% of stillbirths in LMICs occur during labor, highlighting missed opportunities for timely obstetric interventions and underscoring the need to strengthen intrapartum care.[6]

Strengthening antenatal and intrapartum care, timely risk identification, and emergency obstetric services (EmOC) are evidence-backed strategies for reducing preventable stillbirths. Studies show that basic EmOC can reduce intrapartum-related neonatal deaths by 40%, while comprehensive EmOC can achieve a similar reduction. [7] However, in India, these services remain inconsistently available and inequitably accessed. Delayed initiation of ANC, inadequate risk screening, fragmented and uncoordinated referral pathways, and limited availability of appropriate healthcare services, particularly at primary and secondary levels continue to compromise maternal and neonatal outcomes. [8] These challenges are compounded by financial constraints, transport barriers, and persistent knowledge, attitude, and practice (KAP) gaps within communities, which delay timely recognition of danger signs and reduce care-seeking motivation. [9]

National programs such as the India Newborn Action Plan (INAP) and the Operational Guidelines for Sentinel Stillbirth Surveillance aim to improve antenatal care (ANC) coverage, promote skilled birth attendance, and strengthen reporting systems.[10,11] However, their effectiveness remains constrained by a range of socio-behavioral and systemic barriers, including low health literacy, gendered norms, son preference, and inadequate follow-up care. In this context, there is a critical need for deeper insight into the lived realities and care experiences of women and families affected by stillbirth insights that go beyond quantitative indicators and facility-based metrics. [12]

To address this need, verbal autopsy complemented by social autopsy, is increasingly recognized as a valuable tool for community-based approach to examine the underlying social, cultural, and systemic factors contributing to stillbirths. [13] By analysing care-seeking behaviours, healthcare experiences, and structural deficiencies, social autopsy helps unravel the delays and barriers that prevent timely and effective maternal care. The method goes beyond biomedical explanations, capturing lived experiences, missed opportunities, and socio-cultural determinants that shape maternal health and pregnancy outcomes. [13]

This study employs social autopsy to systematically explore the pathways leading to stillbirths and their psychosocial, behavioral, and health system implications for women and families. [14] Specifically, it examines how delayed recognition of complications, inadequate quality of care, gaps in EmOC, and harmful socio-cultural norms and practices contribute to preventable stillbirths. The study also explores post-stillbirth consequences,and continuum of care received among the affected. The findings aim to generate actionable insights that inform context-specific strategies to strengthen maternal healthcare delivery and reduce stillbirths. [14] These insights are intended to inform policy, health worker training, and community engagement strategies aligned with India’s commitment to achieving a single-digit stillbirth rate by 2030.

## Methodology

This study is part of a 36-month, quasi-experimental pre-post design implementation research (IR) project named Strategies to Help in Optimal Pregnancy Outcomes and Reduce Stillbirths in India (SHRiSTI) conducted across seven sites in India.[15] The project aims to develop an optimized delivery model for the effective implementation of evidence-based interventions to reduce stillbirths.[15] This specific study was carried out at one of the seven sites during the formative phase of the IR project, utilizing an exploratory qualitative approach for data collection.[16]

Additionally, as this study was embedded within an ongoing implementation research project conducted in collaboration with the Haryana state and Palwal district health system, Government stakeholders were engaged as the core implementers to support system learning, interpretation of findings, and future co-designing of the implementation strategies. However, they were not involved in participant recruitment, consent processes, or qualitative data collection to avoid any perceived power imbalance.

### Study setting

The Indian national average stillbirth rate (SBR) is 12.4 as per the Health Management Information System (HMIS) 2019−20 reports and the Indian northern belt starting from Himachal and Uttarakhand till West Bengal (WB) through Punjab, Haryana, Uttar Pradesh (UP) and Bihar is found in the intermediate SBR range. [17] The formative phase of the primary implementation research study was conducted between September 2024 and February 2025 in the semi-urban and rural areas of low performing district of Haryana, which is located approximately 100 km away from India’s capital, Delhi. [17] This district has two secondary level of health care, six community health centres and fifteen primary healthcare centres. [17,18] Additionally, 72 health and wellness centres (HWCs) are functional in this area and 890 Accredited social health activists (ASHAs) serving a population of approximately 13,00,000. In 2024, approximately 40% of women delivered in government health facility and 6% home deliveries and rest preferred private health facilities. Around 66% received at least one ANC visit, but only 40% women got registered in first trimester. [17]

### Study participants and sampling strategy

For this study, stillbirths were defined according to the Government of India’s national guidelines [19]. Stillbirth is defined as Gestational age of 28 completed weeks or more, or birth weight 1000 grams or more or body length of 35 cm or more. To identify eligible participants, the research team collaborated with Accredited Social Health Activists (ASHAs) to obtain routine line-lists of adverse pregnancy outcomes between 1st August and 30th September 2024. The list included 60 events labelled as stillbirths and 18 early neonatal deaths (death within 24 hours of birth), resulting in 78 adverse pregnancy outcomes. The data collection for this social autopsy was conducted during the formative phase of the primary implementation research (IR) project, between December 2024–10^th^ February 2025. During sensitization meetings conducted as part of the pre-formative phase, district health authorities and healthcare providers highlighted frequent misclassification of stillbirths, particularly the use of a 20-week gestational threshold and the incorrect classification of very early neonatal deaths, i.e., deaths of newborns before 24 hours, as stillbirths. To address this, a trained research nurse visited all 78 households and verified outcomes using the World Health Organization’s’ 2016 verbal and social autopsy tool and Government of India stillbirth definitions. Of the 78 outcomes screened, 45 met the study definition of stillbirth and were eligible for inclusion. The ASHAs were engaged only to share routine line-lists of pregnancy outcomes maintained as part of the government health system. These lists were used solely to identify potential households where a stillbirth had occurred. ASHAs were not involved in approaching women, confirming eligibility, obtaining consent, or conducting interviews. All household visits, eligibility verification, and participant recruitment were conducted independently by trained research staff employed by the study, who had no service delivery or supervisory relationship with the participants. Additionally, to obtain a more holistic insight, the perspectives of the healthcare providers was also taken into consideration.

Following this preliminary verification of stillbirth cases by the staff-nurse, a multidisciplinary qualitative research team, comprising study investigators (MD/PhD in Public Health), a social scientist (PhD in Anthropology), and field coordinator/s (trained in Public Health with qualifications such as MPH, PhD, or from allied/alternative health systems), reviewed the cases. Participants were then purposively selected to ensure representation across socioeconomic strata and high-risk pregnancy status, enabling a more nuanced understanding of stillbirth pathways across different contexts. The socio-economic status was developed by gathering information on household assets from the head of the household during the household visits and were categorized into three groups: “poor”, “very poor” and “poorest.”

The distribution of high-risk and non-high-risk pregnancies across socio-economic strata in the qualitative subsample was broadly consistent with patterns noted among all 45 confirmed stillbirths and the larger formative dataset. Given the exploratory qualitative design of this social autopsy study, in-depth interviews were not conducted with all 45 confirmed stillbirth cases. Instead, a purposive subsample of women was selected to ensure analytical variation across socio-economic strata and pregnancy risk status. Given the study’s objective of examining health-system and care-seeking failures, high-risk pregnancies were intentionally over-represented to enable in-depth exploration of missed opportunities for prevention and care. This approach is consistent with qualitative methodology, where the aim is depth of understanding rather than representativeness. Sampling was iterative and continued until thematic saturation was achieved.

In addition to in-depth interviews with women who had experienced stillbirth, in-depth interviews were conducted with healthcare providers, including specialist doctors, medical officers, and staff nurses. The research team had key-informant interviews with five healthcare professionals, including staff nurses (03), medical officers (02), and specialist doctor (01), with five to 15 years of experience in the public health system to understand the challenges in delivering quality maternal care

### Data collection and analysis

In-depth and key informant interview guides were developed for each participant group, including women who had experienced stillbirths, healthcare providers, and community members, to explore their perceptions, care-seeking behaviours, emotional responses, and health system interactions related to stillbirths (**Annexure II**). The guides were translated into Hindi, pilot-tested in a non-study population to ensure cultural appropriateness and iteratively refined for clarity and sensitivity to the topic.

Three trained qualitative interviewers, fluent in both Hindi and the local dialect Haryanvi, conducted the interviews. Interviewers underwent a structured training process, which included an orientation to the study objectives, mock interviews, and field piloting of guides outside the study area. Emphasis was placed on building rapport, avoiding leading questions, and remaining sensitive to emotional cues. Interviewers were trained to remain attentive to emotional cues such as prolonged silence, tearfulness, changes in tone, or visible distress. Interviews were paced flexibly, with pauses offered when participants became emotionally overwhelmed, and participants were reminded of their right to stop or reschedule the interview at any time. One study investigator conducted interviews with selected healthcare providers and frontline staff in Hindi or English.

Interviews were conducted at locations chosen by participants, typically women’s homes or health workers’ workplaces, to ensure privacy and comfort. Written informed consent was obtained prior to each interview, and interviews were audio-recorded when permitted. For those preferring not to be recorded, detailed notes were taken and expanded immediately after interview. Interviewers also noted non-verbal cues and contextual observations, such as tone, hesitations, and environmental dynamics.

Debriefing meetings were held daily among the study team to discuss challenges, review emerging insights, and make real-time revisions to the interview guide. This iterative approach allowed the inclusion of emerging themes into subsequent interviews. Data collection continued until thematic saturation was reached, with no new information emerging in subsequent interviews. All audio-recorded interviews were transcribed verbatim and translated into English by trained personnel. The transcripts were reviewed by the principal and co-principal investigators to verify accuracy and ensure meaning was retained, particularly for culturally nuanced expressions.

Data were analyzed using a combination of deductive (framework-guided) and inductive (data-driven) thematic approaches.[20] A preliminary codebook was developed based on the study objectives and the modified Three Delays Model, and additionally the fourth delay highlighting delay in continuum of care after stillbirth.[20] Through repeated readings, additional codes emerging from the data were integrated. Transcripts were coded manually and using NVivo software, with codes organized into categories and themes through constant comparison. Visual mapping tools in NVivo 16 were used to develop a conceptual map of the care-seeking pathways and contributing factors.

To ensure trustworthiness, multiple strategies were adopted. Investigator triangulation involved multiple researchers independently reviewing transcripts and comparing codes to reduce personal bias and increase analytical rigor. [16] Reflexivity was actively maintained interviewers and analysts regularly reflected on their own social identities (gender, caste, education, language, professional background) and how these could shape data collection and interpretation.[21] These reflections were discussed in analytic meetings and documented. Positionality was acknowledged, particularly as some researchers shared regional and linguistic familiarity with participants yet remained outsiders by education and professional role.[22,23] The research team made conscious efforts to build trust and avoid projecting assumptions during interviews.[22,23] Member checking was carried out informally in selected interviews by summarizing responses back to participants at the end of the conversation to ensure accurate representation. These strategies contributed to the credibility and depth of the study and ensured that findings remained grounded in participants’ lived experiences. The manuscript uses the Standard For Reporting Qualitative Research for reporting its findings (**Annexure-I**)

### Ethical approvals and consent of participate

The study protocol was reviewed and approved by the ethics committee of Society for Applied Studies, (SAS) (Approval number- ERC: SAS/ERC/ICMR Stillbirths Study/2024, approval date- January 24,2024). Additionally, consent was obtained from all women and healthcare providers reading out the information sheet to them. Ethics approval included permission to contact potential participants using routine health system records, provided that initial contact, consent, and data collection were conducted by independent research staff and not by frontline or any government health workers. Participation was entirely voluntary, and refusal to participate had no implications for access to routine health services. Participants could withdraw or request to stop recording the interviews at any time.

## Results

The study included total of 30 participants out of which 25 were women who had stillbirths and five were healthcare providers, was included in the study using purposive sampling. Of the 25 women included in the study, 21 had high-risk pregnancies and four had non–high-risk pregnancies. Participants were drawn across socio-economic strata, with representation from the poorest (n = 13), very poor (n = 9), and poor (n = 3) households (Table 1). High-risk pregnancies were represented across all socio-economic categories, enabling exploration of how social vulnerability and clinical risk intersected to shape care-seeking pathways and health-system experiences.

**Table 1:** Distribution of study participants by socio-economic status and high-risk pregnancy status.

Socio-economic status	High-risk cases	Non-High-risk cases	Total
Poorest	12	1	13
Very Poor	7	2	8
Poor	2	1	3
Total	21	4	25

* The socio-economic status was developed by gathering information on household assets from the head of the household during the household visits and were categorized into three groups: “poor”, “very poor” and “poorest.”

### Participant characteristics

The socio-demographic and medical history of mothers who had experienced stillbirths revealed that nearly equal numbers of participants resided in rural and semi-urban areas. The socio-demographic profile of the study population reveals that most of the women (56%) were between 17–25 years of age, with a mean age of 25.28 years (±4.03). Around 76% of women were non-literate, with only 24% having received formal education; the mean (SD) for education was 4.48 (3.67) years. Most of the participants were homemakers (84%) and 12% of them were employed. (Table 2).

**Table 2:** Socio-demographic characteristics of study population.

Characteristics	Total (N = 25)	Mean (±SD)
	n(%)	
*Age (Years)		
17-25	14 (56)	25.28 (± 4.03)
≥ 26	11 (44)	
Education		
No formal education (0 years)	19 (76)	4.48 (± 3.67)
Primary (1–5 years)	4 (16)	
Upper primary and above (>= 6 years)	2 (8)	
Employment status		
Homemakers	21 (84)	
Employed (formal or informal sectors)	3 (12)	
Religion		
Hindu	17 (68)	
Muslim	8 (32)	

*Only those who have visited the school for formal education have been included in the literate category. While those who received non-formal education, i.e., studies in “Madarsa” for a particular language, i.e., Urdu, has been included in the illiterate category.

84% of women had high-risk pregnancies. Preterm births (<37 weeks) accounted for 60% of cases, with an average gestational age of 34.51 weeks (±4.41). Most women had two or more previous pregnancies, with 48% being in their second or third pregnancy and 44% in their fourth or beyond. Most deliveries were normal vaginal births (84%), 16% caesarean sections. Among 84% institutional deliveries, 68% took place in private facilities, and 16% in public hospitals; 16% were home deliveries. Gender distribution of stillbirths was slightly higher for female foetuses (56%) than male (44%). Most stillbirths (80%) were fresh, indicating intrapartum complications, while 20% were macerated, suggesting antepartum foetal demise that may have gone undetected due to poor antenatal monitoring. (**Table 3**)

**Table 3:** Pregnancy-related characteristics of study population.

Characteristics	Total (N = 25)	Mean (±SD)
	Count (%)	
**High-Risk Pregnancies**	21 (84)	
**Gestational age**		
PTB (<37 weeks)	15 (60)	34.51 (4.41)
NTB (≥37 weeks)	10 (40)	
**Gravida**		
Primiparous	2 (8)	
2^nd^ & 3^rd^	12 (48)	
≥ 4^th^	11 (44)	
**Mode of Delivery**		
Normal	21 (84)	
Cesarean Section	4 (16)	
**Place of Delivery**		
Home Delivery	4 (16)	
Private facility	17 (68)	
Public facility	4 (16)	
**Onset of labor pain**		
Spontaneous	9 (36)	
Induced	16 (64)	
**Gender of the Stillborn**		
Male	11 (44)	
Female	14 (56)	
**Stillborn status**		
Fresh	20 (80)	
Macerated	5(20)	

*N = Total Number of Participants; SD- Standard Deviation; PTB-Pre- Term Birth; NTB- Normal Term Birth

### Pathways leading to stillbirth

**Fig 1:**
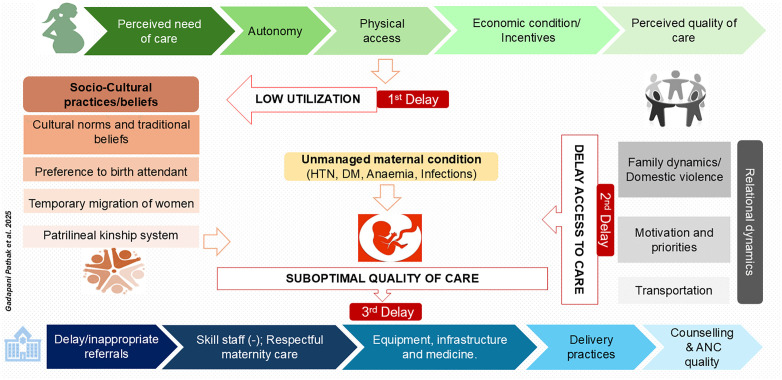
The Three Delay Model.

Through the analysis of participant narratives, several interlinked themes emerged as contributing factors to stillbirth. These were categorized into three major pathways that aligned with the Three Delays Model, highlighting the delays in utilization of healthcare services, delay in reaching healthcare facilities, receiving appropriate and quality care once at a facility. Each of these pathways was shaped by socio-cultural, economic, community, and systemic factors that collectively influenced maternal healthcare utilization and the pregnancy outcome. (**Fig 1**: The Three Delay Model) and detailed description with verbatims are mentioned in **S1 Table**.

### Theme 1: Low Utilization and Access to Healthcare

#### Cultural norms and traditional or supernatural beliefs.

Cultural beliefs play a significant role in influencing pregnancy-related behaviours, particularly in the early months. A widely held belief in many communities is the fear of the “evil eye” (Black eye), which discourages women from disclosing their pregnancy during the first trimester. This reluctance often results in delayed registration and limited access to antenatal care (ANC), in the early months of the pregnancy.

*“In our family, mother-in-law advised me not to share the news of my pregnancy with anyone, not even with our relatives. She believes that disclosing it too early could attract the ‘evil eye,’ which might cause harm or even lead to a miscarriage, especially since I have experienced one before. Following this tradition, I did not reveal my pregnancy during the first four months and only visited the hospital in at the end of my fourth month only.”*

*[* ***Women IDI-2*** *]*

In some minority religious communities within the study area, the practice of lactational amenorrhea, the concept that breastfeeding serves as a natural contraceptive, was identified as a contributing factor to unrecognized pregnancies. Many women assumed they could not conceive while breastfeeding, which led to delayed recognition of pregnancy and short inter-pregnancy intervals, often less than a year. As a result, antenatal care (ANC) registration was sometimes postponed simply because the pregnancy was not identified in its early stages. While these socio-cultural beliefs do contribute to delayed ANC initiation, the research team noted that the overall impact of this factor appears relatively limited at the population level, directing to a broader structural and behavioral barrier, playing a more significant role in hindering timely ANC uptake.

Traditional and supernatural beliefs remain deeply embedded in maternal care-seeking behaviour often shaping pregnancy related decisions. Some women rely on protective rituals and faith-based practices, believing that they can prevent pregnancy complications through spiritual interventions. Common practices include wearing tabeez (amulets) for protection, performing dhuni (incense fumigation) to ward off negative forces, and following strict dietary and activity restrictions prescribed by local customs. These beliefs stem from the perception that pregnancy loss or complications may be caused by supernatural forces, requiring non-medical interventions for protection. Furthermore, some women completely replace medical care with faith-based guidance from religious figures such as Maulvi (priest), instead of seeking professional maternal healthcare. It was noted that these practices offered emotional reassurance but contributed to delay in evidence-based medical care.

“*After my first baby died, I believed it was due to upari chakkar (supernatural forces). During my next pregnancy, I burned Guggul (incense) for purification, but I still lost the baby. I believe wandering paret (spirits) in this area caused the loss. Until I return to my village and seek blessings from a Bhagat (spiritual healer), I will not try to conceive again.”*

*[* ***Women IDI-9*** *]*

#### Patrilineal kinship societal system.

The rural setting revealed the pervasive influence of long-standing patrilineal kinship traditions, wherein male children are prioritized within social, economic, and familial structures. This cultural context significantly shapes maternal care-seeking behavior during pregnancy. It was found that, some families reportedly delay antenatal care (ANC) registration with the desire to determine the foetal sex. While foetal sex determination is prohibited under the Prohibition of Sex Selection (PCPNDT) Act, 1994 in India, respondents indicated that certain families may seek this information from unregistered healthcare providers either locally or in neighbouring states. The Pre-Conception and Pre-Natal Diagnostic Techniques (PC-PNDT) Act is an Indian law aimed at prohibiting sex selection and regulating the use of prenatal diagnostic techniques to prevent female feticide and address gender imbalance. According to some healthcare providers, these practices not only lead to delayed or unregistered pregnancies but may also contribute to selective continuation based on foetal sex. Additionally, the research found that this community follows an androcentric norm. A few women, particularly those with multiple daughters, were found to perceive their pregnancies as unwanted due to family pressure for a male child. As a result, they are less motivated to seek ANC or visit healthcare facilities. In cases where women conceived solely due to family expectations of having a son, they expressed low engagement with ANC services.

“*I didn’t go to the hospital early because I never wanted this pregnancy. I had no desire to have another child. But my husband insisted, saying, ‘We already have four daughters, let’s see, maybe this time it will be a boy.’ Even my parents and family pressured me to carry this pregnancy, so I had no choice.”*

*[* ***Women IDI-15]***

*“Most families don’t get the pregnancy registered in the early months because they want to find out the sex of the baby first... Here, the rules for identification of gender during ultrasonography are strict, so they go to nearby states or some unregistered practitioner to get the ultrasound done and, if it’s needed, they get the abortion.”*

*[* ***Healthcare provider*** *]*

#### Preference for Traditional Birth Attendants (TBAs) and local health practitioner.

Many families prefer traditional birth attendants (TBAs) over institutional healthcare providers. TBAs assure normal deliveries, reinforcing the fear of caesarean-sections. However, in several cases, TBAs misdiagnosed complications as false labour which delayed necessary and timely hospital care. Additionally, women often consult local village non-registered health practitioners for pregnancy-related concerns instead of seeking formal healthcare.

Additionally, in some areas, there is a prevailing misconception in the community that government hospitals favour caesarean sections (C-sections) as a faster alternative to vaginal delivery to reduce staff workload. Most of the women reported that the local community highly stigmatized C-sections, as women who undergo them often viewed as weak or unable to withstand labour pain. Families also fear that C-sections may compromise future fertility, reinforcing a preference for home births with TBA and further deterring institutional deliveries.

*“We first went to a local health practitioner (* ***gaon ki dai*** *, village midwife) in xx village. Despite being advised at the hospital to terminate the pregnancy due to risk to my life, my family believed she could ensure a normal delivery. She checked the baby’s heartbeat, gave me injections and medicines, and I delivered a few hours later…..but the baby was already dead.*

*[* ***Woman IDI-7]***

Additionally, the study team noted that women from the poorest households more frequently described delayed antenatal registration, irregular follow-up, and reliance on informal or unregistered providers, often citing financial constraints, loss of daily wages, and lack of transport as key barriers to timely care.

#### Temporary birth migration.

Research team found some of the women with stillbirth had moved to their maternal house during pregnancy. This is longstanding practice in India for pregnant women to return to their maternal home for delivery and postpartum care and Palwal, Haryana was no different. However, this practice was found to disrupt the regular ANC services as women registered in one district cannot receive services in another. Women who forget or miss-place their Maternal and Child Protection (MCP) card faced additional barriers, as their previous medical records were not accessible in the new location. Furthermore, migrating women miss out on financial incentives under Government of India schemes like Janani Suraksha Yojana/ *Janani Shishu Suraksha Karyakram*, which are tied to their place of registration.

*“In our family, there is this tradition that first child should be delivered in maternal home and my mother also insisted that I should be in maternal home from the early months of pregnancy that is why after the completion of my fourth month I stayed in my maternal home. I did everything and was taking care of me. But when I went there, the hospital refused to register my name, saying my records weren’t available. Since I didn’t have my documents with address of my in-laws place. I was completely fine so did not care much about it”*

*[* ***Woman IDI-9*** *]*

#### Women’s autonomy overpowered by societal norms.

In the study setting, decisions regarding when and where to seek antenatal or delivery care were rarely made by the women themselves. Instead, these choices, such as whether to deliver at a public or private facility, or whether to remain at the maternal or marital home for childbirth, were predominantly influenced or dictated by husbands, fathers or mothers-in-law, or elder male members of the household. Some of the women reported needing permission from male family members to access healthcare services, delaying care even in the presence of complications. Additionally, a few women reported the fear of being blamed for adverse outcomes, particularly in cases of repeated pregnancy loss or the birth of a female child, discouraged them to open discussion and timely health-seeking. So, it was noted that women’s autonomy in healthcare decision-making remained constrained by patriarchal family structures and rigid gender norms in this setting.

*“We live in a joint family, and decisions about check-ups and delivery are taken by elders, especially the mother-in-law. I did not even know where I would deliver until the last month. After my sister lost her baby soon after a hospital delivery, my family blamed the government hospital. Because of this, I chose not to express my preferences and let my husband decide.”*

*[* ***Woman IDI-10]***

#### Low motivation and competing priorities for women.

The research team noted that most of the low-income households often prioritized household responsibilities and childcare over healthcare visits. Many stated that managing domestic tasks and looking after other children took precedence over traveling to health facilities for ANC check-ups. The lack of support from husbands and family members further discouraged them from seeking care, reinforcing the expectation that maternal responsibilities should take precedence over personal health needs. Additionally, a few women with multiple daughters reported that the deliberately postponed ANC registration and healthcare visits. These women expressed low motivation to seek care, citing concerns that once the family discovered they were carrying another female child, they would face mistreatment and emotional neglect. This fear further contributed to delays in accessing maternal healthcare.

#### Economic barriers to care.

Economic constraints were reported as one of the major factors influencing antenatal healthcare utilization among women from low-income households, particularly those where husbands were daily wage labourers, unemployed, or where the woman herself was unemployed. Most of the women reported that the burden of household responsibilities, including managing multiple dependents with a single earning member, often created competing financial priorities and forced them to deprioritize healthcare in favour of sustaining household needs. Many women reported that the potential loss of daily wages due to hospital visits served as a major deterrent, as even a single day’s wage loss had severe financial consequences for their families.

*“My husband is a daily wage laborer who earns daily wages, and if he took a day off to take me to the hospital during pregnancy, we would lose that day’s income. That’s why I avoided going unless there was a serious problem. Even when I wanted to go, we didn’t have enough money for transport, and private hospitals charged too much. The government hospital is free, but they don’t have all the tests, and we would still have to spend money on medicines and scans. So, we just waited and hoped everything would be fine.”*

*[* ***Woman IDI-03*** *]*

In addition to wage loss, out-of-pocket expenses for diagnostic procedures and transportation costs further limited healthcare-seeking behaviour. Advanced diagnostic tests, such as Level II ultrasounds, costing approximately ₹2,500 ($29), were reported to be unaffordable for economically disadvantaged women. The limited availability of such diagnostic services in government hospitals exacerbated this issue, forcing women to either delay necessary investigations or forgo them entirely. Additionally, the cost of private healthcare services, including consultation fees, medications, and laboratory tests, posed further financial challenges, making institutional care inaccessible for many women.

An informal practice known as “*Badhai culture*” was reported by a few women in the community as another financial barrier discouraging institutional deliveries. Families were expected to pay informal financial amounts to government hospital staff, amounting to ₹1,500 ($17) for female childbirth and ₹3,000 ($34) for male childbirth. These additional financial burdens disproportionately affected multigravida and low-income women, deterring them from choosing institutional deliveries due to the associated costs. As a result, many women opted for delayed or alternative maternal healthcare pathways like going to non-registered medical care practitioner or traditional birth attendant, further limiting access to safe and timely obstetric care. Additionally, a few women reported that ASHA workers provided extra support and assistance to certain families, particularly by regularly accompanying them to hospitals or ANC clinics, which they believed was linked to receiving additional incentives. As most of the women belonged to low-income households, they mentioned being unable to offer any extra incentives to ASHAs. Consequently, they perceived a disparity in the level of care received, feeling that families with better financial status received more attention and assistance.

*“My family was happy when I went into labour, but I lost my baby. In the hospital, I saw families giving money to nurses after a healthy birth before the baby was handed over. Sometimes, families give money out of happiness, but in some cases, the nurses themselves ask for more. I kept wondering-if my baby had survived, would my family also have been asked to do the same?”*

*[* ***Woman IDI-04*** *]*

#### Perceived and experienced quality of services in facilities.

The research team noted that women’s perceptions of healthcare quality were primarily influenced by their past experiences with maternal health services, whether from previous pregnancies or interactions with healthcare facilities. Some families also avoided certain hospitals due to previous negative experiences, such as the death of a family member at that facility. Instead, many opted to seek private care, fearing further referrals and inadequate management at government hospitals. Some women recounted harmful and abusive practices during labour and delivery. A few reported being physically restrained, with hands and legs tied and mouths blocked with cotton, purportedly to control their movement during extreme labour pain. Such actions caused distress, breathlessness, and exhaustion, further exacerbating their suffering. A few women also mentioned physical mistreatment, including slapping and forceful pulling of legs by birth attendants during labour. This poor treatment and hostile attitude from healthcare providers resulted in psychological distress, with some women expressing fear and reluctance to seek institutional deliveries, specifically at government set-up, for subsequent pregnancies.

Others mentioned non-compliance with iron and calcium supplements due to nausea, misconceptions about medication safety, and religious beliefs discouraging the use of modern medicine. Dissatisfaction with ASHA workers was also commonly reported, as this lack of active engagement from frontline health workers further weakened trust in public healthcare services.

*“When I delivered my second child at a government hospital, I was sent from room to room and received no help. After multiple tests and long delays, we stopped going back. Because of this experience, I did not visit the hospital even once during my recent pregnancy.”*

*[* ***Woman IDI-13]***

### Theme 2. delay in seeking care

#### Perceived need for Antenatal Care (ANC).

Many women reported not seeking ANC in the early months of pregnancy, believing it was only necessary in the presence of complications. A commonly expressed sentiment was, “*I was not facing any issues, so I didn’t feel the need to visit the hospital and delay it till in occurrence of complication.*” Pregnancy was largely perceived as a natural process that did not require medical intervention, leading women to deprioritize routine check-ups. Familial influence, particularly from mothers-in-law, further reinforced this perception. Several women shared that they were discouraged from attending ANC visits, as elder family members recounted their own pregnancies without medical supervision, reinforcing the belief that ANC was unnecessary unless complications arose. Among multigravida women, a casual approach to ANC was frequently noted. Many assumed that if previous pregnancies had been uncomplicated, subsequent ones would also be problem-free, leading them to forgo ANC visits unless health concerns became apparent. Their confidence in prior successful pregnancies often resulted in missed opportunities for early risk identification and preventive care.

*“I didn’t feel the need to go for check-ups because I had no issues. Pregnancy is natural, my mother-in-law always says that in our time, we never went to the hospital unless something was seriously wrong. So, I also thought it wasn’t necessary.”*

*[* ***Woman IDI-8*** *]*

#### Physical accessibility of appropriate care.

Women residing in rural and remote areas reported significant transportation challenges in accessing healthcare facilities, particularly during antenatal care, labour, and childbirth. Some villages are located over 20 km from the district hospital, making timely access to emergency obstetric care extremely difficult. Several women also mentioned that their villages lacked direct transport connectivity, requiring them to walk or take some manual driven vehicle, for 7–8 km, to reach the nearest main road before they could access public transportation. In addition, a few women mentioned that certain health centers were not well-located geographically and had unpaved roads too. These women faced longer travel times, difficulties during emergency situations, and increased reliance on personal arrangements for transport, which many families could not afford.

*“We don’t have our vehicle and even if we arrange a private vehicle, the roads are so bad that it takes too long to reach the hospital. During the rainy season, it becomes even worse because of mud as my house is in between the fields. Sometimes, I avoided visiting the health facility because the road is full of mud and water.”*

***[Woman IDI-14]***

#### Dependence of the women for transportation.

A major barrier to timely ANC access, as stated by pregnant women, was their dependence on family members and community health workers, particularly ASHAs, for healthcare navigation. Several women mentioned relying on their husbands, other male family members, or ASHA workers to accompany them to health facilities. Many reported that the absence of an accompanying person often led to missed ANC appointments or complete avoidance of maternal healthcare. This dependence, as mentioned by women, highlights their limited agency in seeking care, leading to delays in timely healthcare access.

Another critical barrier identified was the inefficiency of ambulance services in reaching women in need of urgent maternal healthcare. The state-run ambulance service (dial “112”) was reported to be unreliable, often taking a significant amount of time to arrive at the requested location. A major challenge was that the number served as a centralized helpline for all emergency services, making it difficult to connect specifically for ambulance requests. Additionally, the follow-up questions routed through the IVR system often delayed the actual booking process. Additionally, the ambulances used were large vehicles that could not navigate the narrow, poorly maintained village roads, requiring families to physically transport the pregnant woman to a more accessible location where the vehicle was parked. These transportation delays sometimes resulted in childbirth occurring in transit.

*“I cannot go to the hospital on my own. Because I don’t know the way to the hospital, and I have never gone out alone. I can only go if my husband or with my mother-in-law. Once, I was in severe pain, but no one was at home to take me, so I had to endure it the whole night…My husband and mother-in-law decide where I should go and which doctor to see. I just had to follow them.”*

[**Woman-IDI-1]**

#### Community and family dynamics in healthcare-seeking.

Restrictions on movement were also widespread, with women being advised against traveling alone, particularly during the eighth and ninth months of pregnancy. Some women further reported superstitions surrounding water bodies (rivers, ponds, gher-ghat), as stepping outside during late pregnancy was believed to cause premature labor or harm the baby. Collectively, these cultural beliefs led to significant limitations on physical activity during pregnancy and some women reported that even if they felt the need to seek health facilities, they delayed seeking care.

In addition to restrictive practices, some women disclosed experiencing domestic violence, including physical and verbal abuse. Many women who lived in households where domestic violence was prevalent reported feeling fearful and hesitant to communicate pregnancy-related concerns, such as spotting, dizziness, or swelling of the legs to family members and husband. As a result, they often ignored these symptoms and delayed care seeking. But most of these women acknowledged that these could be early warning signs of complications. Sadly, a few women rationalized this mistreatment, stating that their husbands were exhausted from work and should not be blamed for their behaviour, if it did not directly harm the baby. A few women also mentioned being given specific plant stems or liquids to consume, as their families believed that such substances would ensure the birth of a male child. However, some women reported experiencing severe abdominal pain following these practices, which they suspected contributed to their stillbirth. However, these women delayed seeking care from hospitals as family didn’t allow them to do so.

### Theme 3: Inadequate quality of care received at the healthcare facilities

#### Delays and inappropriate referrals.

Women reported negative experiences in government hospitals, particularly multiple referrals during the intrapartum period. Some women reported prolonged delays in receiving appropriate care due to multiple referrals across different health facilities. Many were first referred from Primary Health Centers (PHCs) to Community Health Centers (CHCs) and then to District Hospitals (DHs), often without receiving necessary interventions at the earlier levels. Additionally, most of the women mentioned that the government hospitals have complete lack of respectful maternity care, and they are mistreated during intrapartum and delivery process. For some, the referral process itself was time-consuming, with delays caused by administrative formalities before transfer. A few women mentioned that hospital staff took significant time completing paperwork, further prolonging access to care.

*“During labour, we first went to a gaon ka doctor (village doctor), who checked the baby’s heartbeat and asked for an ultrasound. The government hospital also refused treatment without an ultrasound and referred us elsewhere without explaining anything. By the time we reached the district hospital (DH), we were again referred to a medical college. People believe that once someone is referred to that hospital, the chances of survival are very low. Fearing the outcome, my family decided to take me back home.*

***[Woman IDI-18*** *]*

Most of healthcare staff mentioned that there was only one sub-optimally functioning First Referral Unit (FRU) and most of the Community Health Centers (CHCs) and Primary Health Centers (PHCs) lacked Comprehensive Emergency Obstetric Care (CeMOC) services, while the Basic Emergency Obstetric Care (BeMOC) facilities were not adequately equipped to handle obstetric emergencies. This deficiency in services led to frequent referrals to higher-level hospitals, further increasing delays in emergency care and the burden on women seeking institutional deliveries. Nevertheless, a few nurses and Auxiliary Nurse Midwives (ANMs) mentioned that families consulted religious figures (Maulvis) before deciding to visit the referred facility, leading to additional delays and women reached centers with full dilatation and it was difficult for them to handle such cases specifically during night hours. Most of the women who were referred to medical colleges or higher facilities often did not follow through with the referral due to long travel distances, unfamiliarity with the facility, and concerns about not having family support at these locations. Research team also noted that there was a complete lack of referral linkage between one center and another and in such cases, it was obvious that the families feels lost as they are already in a very precarious state and coming to a new hospital at end moment and not getting any assistance from the health system will make them feel scared and will add to their negative experience during child birth.

*“We referred a woman from the CHC to the district hospital due to her condition, but her family consulted a Maulvi (religious priest), who advised them to stay. They performed kriya (religious rituals) and jhaad-phook (faith-based healing). The family refused referral, and by the time her condition worsened, it was too late.”*

*[* ***Healthcare provider*** *]*

*“We often refer patients because in some centers services for managing high risk conditions are limited, but there is no proper referral linkage. Families are told to go on their own, and many do not reach the next facility in time.”*

*[* ***Healthcare provider*** *]*

High-risk pregnancies across all SES categories reported fragmented referrals and poor counselling.

#### Disorganized and inappropriate services at the healthcare facilities.

Women also reported some additional concerns included excessive documentation requirements, disorganized ANC services, scattered diagnostic and Outpatient Department (OPD) facilities, and the overall difficulty in navigating the system, which made hospital visits burdensome and time-consuming. Women also reported infrastructural inadequacies like lack of proper waiting areas and overcrowded spaces absence of basic amenities including clean drinking water, functional toilets, and cooling facilities, making it non-compliant especially during extreme summers. Women reported absence of structured consultation areas and poorly organized screening facilities contributed to suboptimal patient experiences, with women often forced to stand during outpatient consultations, receiving brief and impersonal interactions with healthcare providers Many also expressed frustrations over the non-availability of diagnostic services, citing limited ultrasound facilities at CHCs and PHCs and restricted access to Level II scans at district hospitals. Diagnostic tests such as Oral Glucose Tolerance Tests (OGTT) and thyroid function tests often required separate appointments, necessitating multiple hospital visits, increasing both financial and logistical burdens. Many women noted minimal assistance in arranging transportation to healthcare facilities and inadequate guidance on ANC services and available resources by ASHAs. A few women reported not receiving any antenatal supplements due to irregular supplies and distribution issues at government facilities.

*“ Going to the hospital is very difficult. We are sent from one place to another for tests, made to stand in long lines, and often asked to return on another day just to collect reports. There is no proper place to sit, and sometimes we wait outside in the heat. After repeated experiences like this, it feels pointless to keep going.*”

*[* ***Woman IDI-6]***

*“ASHA worker only came once during my sixth month. She just noted down some details and left. She didn’t say anything about what I should be careful about or what I should do for my health.”*

*[* ***Woman IDI-5]***

#### Inadequate skill of staff and gaps in clinical management.

Several women mentioned encounters with inexperienced healthcare providers during labour and delivery. A few recalled that the attending staff appeared uncertain in their actions, frequently discussing cases among themselves before proceeding with medical interventions. In one instance, a woman reported experiencing severe pain following an unnecessary per vaginal (PV) examination in the government hospital, after which she noticed an absence of fetal movement. She strongly suspected that mishandling during the PV examination contributed to foetal distress and stillbirth. One woman described how she delivered without medical assistance at a CHC, as the nurse on duty was asleep despite repeated calls from family members. The baby was left unattended for 30–40 minutes, with the placenta still inside and no pain relief provided. Additionally, newborn resuscitation procedures were often delayed, as reported by a few women. In some cases, birth attendants attempted to revive the newborn without proper preparation, using tables cluttered with medical supplies for resuscitation. Women described watching their unresponsive newborns being handled without urgency, leading them to believe that efforts to save the baby were merely performative rather than genuine attempts to provide emergency care.

The research team also noted that there was absence or lack of appropriate counselling skills among the staff. Many women received no counselling regarding high-risk pregnancy (HRP) factors, despite attending antenatal check-ups. Several reported that although they had been categorized as high-risk, healthcare providers failed to explain the implications or offer guidance on dietary changes, lifestyle modifications, and follow-up care. A few recalled the term “high-risk” mentioned during consultations, but without context or explanation, leaving them uninformed about how to manage their condition. Some women left facilities with prescriptions for gestational diabetes or high blood pressure, but without clear instructions on medication adherence or lifestyle adjustments, further increasing their vulnerability to pregnancy complications. Additionally, there was no structured approach to educating women about ANC at the community level. Many ASHA workers, ANMs, and AWWs did not provide any guidance on the significance of monitoring pregnancy-related risks. Some women mentioned that ASHAs visited their homes only for documentation purposes, without offering meaningful health advice or emotional support.

“*At the government hospital, no senior doctor was present at night. Young nurses examined me roughly without proper explanation, causing severe pain. Soon after, my baby’s movement* *stopped. Later, a* ***madam*** *(senior staff) examined me again and then said the baby had died, without explaining what happened*.”

*[* ***Woman IDI-17*** *]*

#### Infrastructural and equipment deficiencies.

Many women described inadequate medical infrastructure as a significant barrier to quality maternal care. Some reported that critical diagnostic tools, such as ultrasounds, were either unavailable or required multiple hospital visits due to restricted access at government hospitals. A few mentioned that financial constraints prevented them from undergoing Level II scans, which are essential for detecting fetal abnormalities.

Frequent power outages and lack of power backup in some CHCs and PHCs further compromised care, especially for newborn resuscitation. Some healthcare staff reported that in certain cases, essential equipment such as warmers and Ambu bags were non-functional, delaying crucial interventions. A few healthcare staff acknowledged that these compromised the quality of care and reinforced negative perceptions of public healthcare facilities, prompting many women to seek care from private hospitals or forego institutional healthcare altogether.

“*The reality is, we don’t even have basic facilities here. There’s no baby warmer, no power backup, no ventilators, and, most importantly, no pediatrician or gynecologist. We try our best, but when things go wrong, the families hold us responsible. They say, ‘Because the government hospital lacks facilities, our baby died.’ But what can we do? We don’t have the resources to save such critical cases.”*

***[Healthcare provider]***

*“The hospital asked me to get an ultrasound, but the machine was unavailable, and I was sent elsewhere. Because scans were costly and done only on certain days, I could not complete them. Later, I was told that earlier detection might have helped my baby, but I never got that chance”*

***[IDI-10]***

The research found that the intersection of socio-economic vulnerability and high-risk pregnancy appeared to exacerbate delays, with women from poorer households with high-risk pregnancies experiencing compounded barriers related to affordability, mobility, and weak referral support*.*

Additionally, we examined both the continuity of postpartum care among women who experienced stillbirth and the broader implications of stillbirth on women’s health.

### Theme 4: Lack of postpartum continuum and bereavement care

Despite the profound physical and emotional toll of stillbirth, no structured follow-up care or bereavement support was provided to affected women with stillbirths. This “fourth delay” reflects a critical breakdown in the continuum of maternal care. Several interrelated factors contributed to this gap:

#### Absence of postpartum and bereavement care plan and procedures at the health-system level.

Most of the healthcare providers reported not having any clear clinical or operational guidelines for managing women after a stillbirth. Unlike live births, stillbirths were not followed by scheduled postnatal check-ups, referrals, or counselling sessions. Many health workers appeared ill-equipped or hesitant to address bereavement, possibly due to limited training in empathetic communication, grief counselling, or mental health screening.

Affected women reported that no formal follow-up care was provided after the stillbirth, either by facility-based providers or community health workers.

Stillbirths often remained underreported or misclassified, which meant women were not flagged for follow-up services in routine registers or maternal tracking systems. Once discharged, women who experienced stillbirths were not proactively followed up by ASHAs, ANMs, or Anganwadi workers, due either to lack of protocols or the assumption that no further care was needed. The research team also noted there was no formal mechanism to screen or support women after perinatal loss in most public health programs.

*There are clear protocols for postnatal care after a live birth, but after a stillbirth, guidelines are not very specific. So, once the woman is discharged the follow-up…… counselling, or even when should the woman be visited remains unspecific*

(***Healthcare professional***)

*“Once the stillbirth happened, everything just stopped. They closed my file at the hospital, and after that no one from the health system contacted me. It felt like because the baby didn’t survive, my care also didn’t matter anymore”*

***[Women IDI-22]***

*“If the baby is stillborn, the case is recorded only for reporting. Health system don’t have a mechanism to flag women after stillbirth for home visits. ASHAs usually focus on mothers with live babies.”*

***(Healthcare provider)***

#### Impact of absence of bereavement support.

Most of the participants mentioned receiving no home visit, counselling, or guidance on managing postnatal complications by the ASHA workers. Some of the women reported reliance on traditional practices and anecdotal advice from family members while some accessed over-the-counter medications or consulted local traditional care providers, they expressed confusion and anxiety over the safety of these treatments, especially regarding future fertility. Additionally, most of the women emphasized that they received no instructions from ASHAs, ANMs, or Anganwadi workers, and many were unaware of how to manage lactation following a stillbirth.

*“After the stillbirth, my chest became hard and very painful. I was afraid to take medicines to stop the milk, so I followed traditional remedies suggested by my mother-in-law, like wearing an undershirt inside out. After a few days, the milk dried up, and I felt that the remedy had worked* ***.”***

***[Woman IDI-18]***

In addition to physical symptoms, women reported intense grief, anxiety, and recurring thoughts about their loss, yet none received any form of bereavement counselling or emotional support from the health system. Many women described feeling isolated, overwhelmed, and blamed by family members. One woman shared, *“Everyone blames me. They keep saying it happened because of me… but I know I did nothing wrong.”*

Several women who had lost male children or experienced multiple stillbirths described a deep sense of worthlessness and abandonment, particularly in households where sons are culturally preferred. *“If my baby had survived, I would have mattered… but now, without a child, I have no worth,”* stated one mother. For some, the fear of future pregnancy loss led to chronic anxiety. Women expressed ongoing worries about conceiving again, questioning whether the next pregnancy would also end in loss. As one noted, *“I keep worrying, will I ever have a child? If I do, will they survive?”* These emotional burdens were intensified by social stigma and the absence of structured mental health support.

#### Lack of awareness of need for care and entitlement among affected women.

The research team also noted that even among women who were employed in government jobs, there was a lack of awareness regarding basic entitlements after stillbirth. One government staff reported receiving only 45 days of paid leave following her stillbirth, on compassionate grounds. She reported experiencing complications and requirement of a longer recovery period was necessary. However, any leave taken beyond the 45 days was unpaid, placing her in a difficult position where she had to choose between her health and financial stability. Another woman, working as a house help, reported that she was denied any form of leave after experiencing a stillbirth. Her employer dismissed her need for rest, implying that recovery was only warranted if the baby had survived. She was pressured to return to work within a week and received multiple calls questioning the legitimacy of her absence.

*“My supervisor madam gave me 45 days of leave, but I didn’t know whether it was official or not. Usually, maternity leave is for six months, but I was only given 45 days. My case was a bit complicated, so I needed more rest. That’s why I extended my leave for three more months, but all the leave after the 45 days was unpaid. I don’t even know if I got my full rights or not… but I had no choice but to take leave without pay*”

***[Women IDI-16]***

*“My madam didn’t give me leave. I only got one week off, but she kept calling me again and again. She said, ‘Why do you need leave? Your baby didn’t survive, why do you need a whole month of rest?’ They thought that leave is only needed if the baby is alive. But my health was also suffering… I was weak, I was grieving. But no one understood that I needed rest too.”*

***[Women IDI-24]***

### Theme 5: Implications of stillbirth on women

Beyond disruptions in care pathways and health-system delays, the social autopsy revealed that the experience of stillbirth had profound and enduring consequences for women’s emotional well-being, physical health, family relationships, and social lives. These impacts extended well beyond the immediate loss of the baby and shaped women’s postpartum experiences, future reproductive decisions, and interactions within households and communities. Detailed findings are presented in **S2 File**. The following subthemes describe the multifaceted implications of stillbirth on women’s lives.

#### Deep grief and constant thoughts about the loss.

Most women described profound emotional distress after losing their babies. Several reported shock and an inability to process the event, leading to persistent grief. A few women who had lost male children expressed experiencing intensified sorrow, as their families placed higher value on male offspring. In some instances, families concealed the death of a male child from the mother to protect her from emotional distress, but this often resulted in greater feelings of detachment and confusion.

Some women mentioned having persistent thoughts and worries about the stillbirth. They frequently reflected on the efforts they had made during pregnancy, the complications they endured, and the financial burden their families faced, questioning why their baby did not survive despite all these sacrifices.

*“I already have four daughters, and now, even this boy is gone… I do all the work, but my head starts hurting whenever I think about it. I keep wondering, if God had just given me a healthy baby, there would be no worries. But what can I do now? I am just exhausted.”*

For some women, stillbirth deepened fears regarding future pregnancies, particularly in cases of recurrent pregnancy loss. One woman reported constant nervousness about whether her next pregnancy would also end in loss, especially if she conceived another male child. This apprehension about future pregnancies and child survival led some women to hesitate before conceiving again.

#### Physical health issues.

Several women experienced breast engorgement (extreme pain and swelling due to milk retention) following stillbirth. Women reported tightened veins, severe discomfort, and fever due to the condition.

To manage this, some women followed traditional practices, such as wrapping their breasts with their husband’s towel (Pati ka Angocha) for four days to two weeks to stop milk production. A few women mentioned wearing their husband’s vest inside out or reversing their own innerwear as part of cultural healing methods.

Medical interventions included taking prescribed medication from local village doctors or hospitals. Some women with young children fed them the retained breast milk, while others used breast pumps to relieve discomfort.

*“When my milk started coming in, I used a machine to express it. But my mother and mother-in-law told me not to take out all the milk. They said to leave some in my breasts, or else if the baby’s soul lingered, the milk would keep coming. No one, no ASHA, no Anganwadi worker, ever told me anything about what to do. Since I wasn’t taking all the milk out, my chest felt heavy and painful for two or three days. But after that, it just dried up.”*

#### Reduced family care and social isolation.

A few women reported a noticeable decline in care and attention from their families after experiencing a stillbirth. Some mentioned feeling ignored or neglected by their household, with family members no longer prioritizing women’s well-being as they would have had the baby survived.

Several women also faced stigma and avoidance from neighbours and community members. One woman shared that her neighbours stopped responding to her greetings and actively avoided conversations, leaving her feeling isolated. This social exclusion led some women to withdraw from social gatherings and public interactions to avoid judgment and scrutiny.

*Earlier, people would respond when I greeted them with* ***‘Namaste’ or ‘Ram-Ram’*** *(traditional greetings). After losing my babies, they stopped speaking to me. People say, ‘She kills her babies,’ and because of this, I no longer go out or talk to anyone.”*

***[Woman IDI-16]***

In many households, stillbirth was not recognized as a significant medical or emotional event. Families often expected women to “move on” quickly, resuming household chores without emotional processing or recovery time. Women were often expected to prioritize family duties over their own recovery, with little consideration for their mental or physical well-being. Some women reported being avoided or blamed by neighbours and relatives, further disincentivizing disclosure or care-seeking.

#### Blame and domestic violence.

Some women reported being blamed by their families and communities for the loss of their baby. These women internalized feelings of guilt and helplessness, believing they were responsible for their child’s death. A few women also experienced domestic violence or harsh treatment from family members following the stillbirth. One woman mentioned that the abuse intensified after her loss, as her family believed the child’s survival would have prevented conflicts. In some cases, the loss of a male child heightened tensions, leading to emotional and physical distress for the bereaved mother.

*“They dragged me out of the house, holding my hand. At that moment, I was so furious, I didn’t know what I was saying… and then I left. All of this is happening because the baby is gone. If my baby had survived, I would have mattered… but now, without a child, I have no worth.”*

***[Woman-IDI 23]***

#### Reliance on traditional practices after experiencing stillbirth.

Several women relied on traditional and supernatural practices to safeguard future pregnancies. A few women visited spiritual healers (Hafiz or Baba) for protective measures, receiving amulets, written prayers (nakkash), and herbal remedies. A few described rituals such as burning spiritual writings and inhaling the smoke for purification, while others chose complete isolation, staying indoors throughout pregnancy to avoid exposure to harmful external influences. Fear of supernatural entities such as upari chakkar (evil spirits), paret (ghosts), and buri aatma (malicious spirits) was deeply ingrained. One woman believed she lost her baby due to wandering spirits near her home, leading her to perform protective rituals before planning her next pregnancy. Another postponed conceiving until she could return to her village and undergo traditional spiritual cleansing. While some women simultaneously sought medical interventions, family influence often encouraged blending traditional and clinical care, reinforcing the interconnectedness of cultural beliefs and maternal health practices.

## Discussion

This study explains the complex web of individual, systemic, and socio-cultural factors that contribute to stillbirth in rural North India. Through a social autopsy lens, the findings offer a detailed understanding of missed opportunities across the continuum of maternal care, from delayed pregnancy recognition to intrapartum complications, and highlight the interplay between community practices and structural health system limitations.

Low awareness about the ANC and a perceived lack of need were prominent drivers of delayed or absent ANC utilization. Socio-cultural beliefs around the “evil eye,” rituals to protect the fetus, and spiritual causation of complications contributed further to poor healthcare-seeking behavior. While these practices may provide emotional security, they divert women from evidence-based care. In particular, the use of spiritual healers (e.g., Maulvis, Bhagats) instead of formal providers illustrates the pluralistic health-seeking landscape, where biomedical and non-biomedical logics coexist, a phenomenon also documented in Bangladesh.[24] Socio-cultural dynamics, including gendered norms, low decision-making autonomy, and preference for male children, further restricted women’s access and motivation for timely care. In some cases, traditional practices such as reliance on midwives, spiritual healers, or unverified home remedies delayed access to biomedical interventions. The integration of such practices with clinical care, often driven by family elders, reflects the pluralistic nature of healthcare-seeking in rural India and poses both a challenge and an opportunity for culturally sensitive interventions.

Timely ANC registration and regular follow-ups are essential for identifying high-risk conditions such as gestational diabetes, hypertensive disorders, and foetal growth restriction, all significant contributors to stillbirth. However, many women only sought care when complications became evident, reflecting a deeply rooted belief that pregnancy is a natural, uneventful process unless symptoms occur. This perception significantly hinders early and proactive engagement with maternal health services. This perception was reinforced by intergenerational narratives, particularly from mothers-in-law, normalizing pregnancies without medical supervision. Similar observations have been reported in Afghanistan, where maternal health is shaped more by tradition than biomedical guidance, resulting in low health literacy and poor engagement with ANC services.[25] Biologically, this delay critically limits the timely detection and management of conditions such as gestational diabetes, preeclampsia, or fetal growth restriction, all of which are known precursors to stillbirth.[26] Additionally, the delays in reaching care were underpinned by infrastructural, geographical constraints and socio-cultural norms. Poor road conditions, unreliable ambulance services, and the long distance to referral centers created formidable physical barriers. However, even more salient where the social ones were that most women reported being unable to travel alone, and access to care was contingent on the availability of male companions or family permission. These findings reflect structural gender inequality and echo similar patterns of constrained female mobility noted in other South Asian contexts.[27] The notion that pregnancy-related decisions rest with male family members severely compromises timely care, especially during obstetric emergencies.

Inadequate counselling on high-risk pregnancy status further exacerbated the problem. Many women remained unaware of their elevated risk and the steps required to mitigate it. Without adequate health literacy, women lacked the capacity to navigate the health system, make informed decisions, or adhere to medical advice. From a biological standpoint, unmanaged conditions like preeclampsia or anaemia can lead to placental insufficiency, foetal hypoxia, or intrauterine growth restriction, common causes of late or intrapartum stillbirths.[8] This gap in risk communication reflects broader deficiencies in provider, client interactions, as reported in maternal health literature across India and sub-Saharan Africa.[6,25]

At the point of care, delays in receiving appropriate, respectful, and timely treatment were strikingly evident. Nearly all women who delivered in public facilities recounted experiences of neglect, verbal abuse, or chaotic referrals. Some described being examined by staff without explanation or consent, contributing to both psychological trauma and missed clinical cues. The reported instances of repeated per-vaginal examinations, lack of birth attendants, and delayed resuscitation are indicative of systemic neglect. Biologically, prolonged or obstructed labour without timely intervention can result in foetal hypoxia, while ineffective neonatal resuscitation may lead to neonatal demise misclassified as stillbirth. These preventable factors highlight a failure to adhere to evidence-based clinical protocols.[28,29]

Systemic gaps in service delivery, particularly at the primary and secondary levels, resulted in multiple referrals without definitive care. Most of the tertiary and secondary level health care lacked gynaecologists, paediatricians, or emergency equipment, violating the basic requirements of Basic and Comprehensive Emergency Obstetric Care (BEmOC/CEmOC). Delays due to inappropriate referrals, unavailability of staff, or administrative bottlenecks directly contributed to intrapartum stillbirths. These gaps of the staff shortages, non-functional equipment, and inappropriate skills reflect not just capacity constraints but breaches in clinical and ethical standards. These systemic failures may directly lead to stillbirths via delays in labor monitoring, poor fetal distress management, or inadequate resuscitation. Similar findings have been documented in sub-Saharan Africa, where disrespect and mistreatment are linked to a major determinant of poor perinatal outcomes.[30]

Postpartum neglect, representing the “fourth delay,” emerged as a critical gap. Despite the profound physical and emotional burden of stillbirth, none of the women received structured follow-up, lactation support, or bereavement counselling. Breast engorgement was self-managed using cultural practices in the absence of medical advice. Emotional distress, including anxiety, grief, self-blame, and stigma, was universal but unacknowledged by the health system. The complete absence of psychosocial support reflects a systemic blind spot: maternal health programs often end at childbirth, failing to address grief or trauma, particularly when the baby does not survive. This is consistent with global reviews that emphasize the invisibility of stillbirth within postnatal care frameworks.[31,32] Additionally, affected women, even those who were educated and employed, were often unaware of their legal entitlements. According to the National Health Mission, Haryana (Memo No. NHM/Admn/Misc./2022/8725–46), women who experience a stillbirth are entitled to 45 days of paid leave.[33] In contrast, the Ministry of Personnel, Public Grievances & Pensions, Government of India, issued a directive in September 2022 providing for up to 60 days of leave in such cases.[34] This discrepancy reflects a lack of harmonization in entitlements across states and limited awareness regarding these provisions. Given that the biological and emotional consequences of stillbirth are universal, inconsistencies in maternity leave policies warrant greater attention and policy standardization.

The intergenerational consequences of stillbirth, particularly on women’s mental health and social standing, were equally evident from the findings of this study. Several women described being blamed for the death, facing social exclusion, and experiencing domestic violence. The loss of a male foetus amplified their marginalization within patrilineal family structures. In patriarchal contexts, where women’s value is linked to childbearing and especially to birthing sons, stillbirth becomes not just a medical event but a sociocultural rupture with long-term implications. These findings mirror studies from India and Pakistan where son preference directly influences care-seeking and emotional outcomes. [35,36] The lack of bereavement counselling further compounded the psychological burden, leaving women to cope in isolation. Evidence suggests that postpartum mental health support, including grief counselling and peer support networks, can significantly improve recovery, underscoring the need for integrating mental health into routine maternal care.[37,38]

### Implications for policy and practice

Addressing the underlying determinants of stillbirth requires a multi-sectoral approach that integrates health system strengthening, behavioral change interventions, and financial protection measures.

Strengthening antenatal and intrapartum care is essential to ensure early risk detection, improved patient counselling, and timely management of complications. Expanding high-risk pregnancy screening through community-based outreach programs and increasing healthcare provider training in risk-based ANC counselling can help address gaps in maternal health literacy and improve care-seeking behaviours.

Enhancing referral and emergency care systems is equally crucial. Establishing structured referral mechanisms between PHCs, CHCs, and district hospitals would improve care continuity and reduce unnecessary facility transfers. Investments in emergency obstetric care (CEmOC) at CHCs, coupled with community-based emergency transport models, could significantly reduce delays in reaching appropriate care. Ensuring respectful maternity care and healthcare accountability is key to reducing mistreatment and improving facility-based delivery experiences. Implementing training programs for healthcare providers to improve patient-provider communication and ethical labour practices can address disrespect and abuse during childbirth. Additionally, maternal feedback systems can enhance accountability in facility-based care, ensuring that women’s voices and concerns are integrated into healthcare quality improvement initiatives. Addressing socioeconomic and cultural barriers through financial protection schemes and community-driven behaviour change programs can help bridge existing inequities in maternal healthcare access. Streamlining the conditional cash transfers or introducing voucher programs to offset out-of-pocket healthcare costs would facilitate greater institutional care-seeking, particularly among economically marginalized groups. Engaging religious leaders, traditional healers, and community elders in maternal health interventions could help shift harmful gender norms and promote evidence-based pregnancy care.

Providing post-stillbirth psychosocial and bereavement support is critical to improving maternal mental health outcomes.[39] Integrating bereavement counselling and grief support networks into existing maternal healthcare services would provide essential emotional support to women coping with pregnancy loss. Formalizing post-stillbirth mental health interventions within ANC and postpartum care models can mitigate long-term psychological distress and improve subsequent pregnancy outcomes.

### Strengths and limitations

A key strength of this study lies in its use of social autopsy to capture rich, nuanced narratives around stillbirth from both women and healthcare providers. By exploring experiences across the care continuum, including postpartum consequences, this study offers a holistic understanding of the multifactorial contributors to stillbirth and its implications on women’s lives. The triangulation of perspectives, the use of local dialects, and daily debriefing sessions enhanced the trustworthiness and cultural sensitivity of the findings. Moreover, the study’s application of the Three Delays Model extending to capture the fourth delay, i.e., continuum of postpartum care after stillbirth, provided a robust theoretical lens for identifying health system, behavioral, and community-level bottlenecks.

However, the study has some limitations. First, as a qualitative study based in a single district of Haryana, the findings may not be generalizable to all settings in India. While efforts were made to include women from diverse socio-economic backgrounds, those from extremely remote or marginalized communities may still be underrepresented. Second, some interviews with healthcare providers were not audio-recorded due to privacy concerns, which may have limited the richness of those narratives. Finally, the absence of verbal autopsy data limits the ability to triangulate clinical causes with social factors, which could have strengthened the interpretation of certain findings. Despite these limitations, the study offers important insights that can inform targeted interventions, policy refinement, and future mixed-methods research on stillbirth or implementation research using principles of implementation science [40] in similar low-resource settings.

## Conclusion

Stillbirth remains a deeply rooted public health challenge, influenced by delayed care-seeking, inadequate facility-based services, and socio-cultural constraints. This study underscores the need for health system reforms that prioritize timely ANC, structured referral pathways, and respectful maternity care while addressing economic and cultural barriers to care-seeking. Future efforts should focus on scalable, context-specific intervention models that integrate facility-level improvements with community-driven behavioral change strategies, ensuring that maternal healthcare is accessible, equitable, and responsive to women’s needs.

## Supporting information

S1 Table Findings of thematic analysis based on the social autopsy. (DOCX)

S2 File Summary of the implications of stillbirths on women. (DOCX)

## Abbreviations

ANC: Antenatal Care
ANM: Auxiliary Nurse Midwife
ASHAs: Accredited Social Health Activists
BeMOC: Basic Emergency Obstetric Care
C-sections: Caesarean Sections
CHCs: Community Health Centers
DHs: District Hospitals
EmOC: Emergency Obstetric Care
FRU: First Referral Unit
HMIS: Health Management Information System
HRP: High-Risk Pregnancy
HWCs: Health and Wellness Centres
ICMR: Indian Council of Medical Research
INAP: India Newborn Action Plan
IR: Implementation Research
JSY/JSSK: Janani Suraksha Yojana / Janani Shishu Suraksha Karyakram
KAP: Knowledge, Attitude, and Practice
LMICs: Low- and Middle-Income Countries
MCP: Maternal and Child Protection
OGTT: Oral Glucose Tolerance Test
OPD: Outpatient Department
PCPNDT Act: Pre-Conception and Pre-Natal Diagnostic Techniques Act
PHCs: Primary Health Centers
PV: Per Vaginal
SBR: Stillbirth Rate
TBAs: Traditional Birth Attendants
