# Supplementary material for: Exploring pathways leading to stillbirths and gaps in postnatal care among affected women in a rural north Indian district: A qualitative study using the social autopsy lens
Source: PLoS One. 2026 May 13;21(5):e0347994. doi: 10.1371/journal.pone.0347994 (PMC13170853; doi:10.1371/journal.pone.0347994)
Supplement: S1 Table — (DOCX) [file pone.0347994.s001.docx]

**TITLE:** Exploring pathways leading to stillbirths and gaps in postnatal care among affected women in a rural north Indian district: A qualitative study using the social autopsy lens

Barsha Gadapani Pathak^1,2^*, Sonia Maurya^1^, Shruti Bisht^1^, Pranay Vats^1^, Reema Mukerjee^3^, Vinod Kumar Anand^1^, Sarmila Mazumder^1^

1 Society for Applied Studies, New Delhi, India

2 Centre for Intervention Science in Maternal and Child Health, Centre for International Health, Department of Global Public Health and Primary Care, University of Bergen, Bergen, Norway

3 Division of Reproductive, Child Health and Nutrition, Indian Council of Medical Research, New Delhi, India

***Corresponding author:** Barsha Gadapani Pathak, University of Bergen, Bergen, Norway and Society for Applied Studies, New Delhi, India

Email: [Barsha.Pathak@student.uib.no](mailto:Barsha.Pathak@student.uib.no) and [barsha.pathak@sas.org.in](mailto:barsha.pathak@sas.org.in)

**S1 Table: Findings of Thematic Analysis Based on the Social Autopsy**

**S1 Table: Findings of Thematic Analysis Based on the Social Autopsy**

| **Themes** | **Sub-Themes** | **Verbatim** |
| --- | --- | --- |
| **Low Utilization and Access to Healthcare** | **Cultural Norms and Traditional or Supernatural Beliefs** | *"I shared my pregnancy information with the ASHA worker only after four to five months. No one had asked me about it earlier, so I didn’t feel the need to disclose it myself. Besides, my previous children were born without any medical care, and all are healthy, so I didn’t see any reason to inform them early."*  *"In our family, my mother-in-law advised me not to share the news of my pregnancy with anyone, not even with our relatives. She believes that disclosing it too early could attract the ‘evil eye,’ which might cause harm or even lead to a miscarriage, especially since I have experienced one before. Following this tradition, I did not reveal my pregnancy during the first four months and only visited the hospital in at the end of fourth month.”*  *"When I was four months pregnant with my first baby, I believe I lost the pregnancy due to supernatural forces. Before my miscarriage, I used to burn “Guggul” (incense) for purification. One day, while performing a ritual suggested by a neighbour for better crops in our field, I suddenly felt something entered in my body. I got scared and ran inside my home, and the very next day, I lost my baby. I strongly believe that I have lost my recent pregnancy also due to the “Upari chakkar” (supernatural forces), as this area is surrounded by forests where wandering “Paret” (spirits) can cause harm. If I were in my village, I could have sought help from a “Bhagat” (spiritual healer), but there is no one here. That’s why I am not trying for a baby right now……I won’t conceive until I return to my village and get blessings. In the past, when my children named xx and xy, were born safely, it was only because I had sought healing from a “Bhaktini” (female spiritual healer). I have experienced divine possessions before and believe spiritual interventions like “Tabeez” (amulets) and “Dhuni” (sacred fire rituals) are necessary to protect future pregnancies."*  *"I lost my baby because of “Jinn ki harkat” (supernatural interference), but it was “Allah ka Hukum (God’s will). When my pregnancy was confirmed, I also took medicine from the “Maulvi” (Traditional Healer). I visited him in my 3rd, 5th, 6th, and 8th months. When the first time I went, he tied a “Tabeez” (amulet) around my neck and waist, instructing me to remove it only when labor pain began. The “Tabeez” was meant to protect my baby and prevent any harm. He also provided me “Nakkash Dhaga” that I had to tie around my belly to prevent the pre-term birth. Each time I visited, he gave me “Nakkash” (paper pieces with spiritual writings) to dissolve in water and drink for protection.”* |
|  | **Patrilineal Kinship Societal System** | *"I didn’t go to the hospital early because I never wanted this pregnancy. I had no desire to have another child. But my husband insisted, saying, ‘We already have four daughters, let’s see, maybe this time it will be a boy.’ Even my parents and family pressured me to carry this pregnancy, so I had no choice."*  *"When there were complications with the baby, I felt deeply upset. Everyone in the family was saddened by the loss but spoke to me with care. I was informed about it later. Though the newborn was a boy, my family told me it was a girl, describing her as weak and unhealthy. I already sensed that the baby was gone, but they didn’t reveal the truth right away. To protect me from excessive grief, they reassured me, saying, ‘It was a girl… she was weak… it’s okay.’ They believed that if I had known earlier that I had lost a boy, it would have been even harder for me to cope, and it might have affected my health."*  *“When there were complications with the baby, I felt deeply upset. Everyone in the family was saddened by the loss but spoke to me with care. I was informed about it later. Though the newborn was a boy, my family told me it was a girl, describing her as weak and unhealthy. I already sensed that the baby was gone, but they didn’t reveal the truth right away. To protect me from excessive grief, they reassured me, saying, ‘It was a girl… she was weak… It’s okay.’ They believed that if I had known earlier that I had lost a boy, it would have been even harder for me to cope, and it might have affected my health.”*  ~Staff Nurse  “*If we had told her (respondent) directly that it was a boy, it would have been too difficult for her to handle. That’s why we didn’t say it was a boy, if it had been a girl, we would have told her right away. She kept asking us, ‘What happened? Please tell me.’ So, we just told her that the baby was in the incubator (NICU) to comfort her. Because if one lost a male child then grief is bigger”*  ~IDI-3, Sister of Respondent |
|  | **Preference for Traditional Birth Attendants (TBAs) and Local Health Practitioners** | *“I once called a Dai (traditional birth attendant) when my baby got stuck to one side. This happened during my fifth month……I was experiencing abdominal pain. We called the Dai right away as she lives nearby……When the Dai came, she simply moved my belly with her hands, and the pain went away. She didn’t give me any medicine…… may be adjusted the position of the baby with her skill. There aren’t many Dais around here, and she doesn’t assist with deliveries……….but she has knowledge about childbirth, which is why we sought her help."*  *“We didn’t go straight to a doctor but instead visited a local health practitioner in XX village. My family members advised that I should visit her because when during my ultrasound scan, the doctor informed us that my life was at risk, and I needed to terminate the pregnancy. He warned that if I didn’t, the infection would spread inside me. My family knew about this local health practitioner, and they said she would help us in normal and safe delivery. She checked my baby’s heartbeat and assured me that she would perform a normal delivery. She then gave me an injection and inserted medicine inside my body twice, once at 2 PM and again at 4 PM and I delivered in 2 to 3 hours, but my baby was already dead.”* |
|  | **Temporary Birth Migration** | *“In our family, there is this tradition that first child should be delivered in maternal home and my mother also insisted that I should be in maternal home from the early months of pregnancy that is why after the completion of my fourth month I stayed in my maternal home. I did everything and was taking care of me. But when I went there, the hospital refused to register my name, saying my records weren’t available. Since I didn’t have my documents with address of my in-laws place. I was completely fine so did not care much about it”*  *“After going to my maternal home, I didn’t receive any financial assistance. I had heard that the government provides money for deliveries, but since my delivery happened at my maternal home, the aanganwadi worker told me that money would only be given where I was originally registered in my early pregnancy and if I had taken care from there. But I wasn’t there, so I didn’t get any amount.”* |
|  | **Women’s autonomy overpowered by societal norms** | *“I wanted to go to the hospital when I felt uneasy, but my husband said it was not necessary unless the pain becomes unbearable."*  *“We live in a joint family…… and decisions about delivery or any monthly check-up are taken by elders, not by us….. like by mother-in-law said she never went for monthly check-up when she was pregnant! ……even I didn’t even know in which hospital I would deliver until the last month.”*  *“My sister lost her child after 2 days of delivery in the hospital….. Now my family says it happened because she insisted on going to the government hospital…. Observing all this I choose not to speak about my preferences…… let my husband decide…..”*  *“I cannot go to the hospital on my own. Because I don’t know the way to the hospital, and I have never gone out alone. I can only go if my husband or with my mother-in-law. Once, I was in severe pain, but no one was at home to take me, so I had to endure it the whole night…My husband and mother-in-law decide where I should go and which doctor to see. I just have to follow them.”*  *“Once, I said I needed to go for a check-up in 5th or 6th month, but no one at home was available to take me. I couldn’t go alone. I asked the ASHA worker, but she said she couldn’t arrange a vehicle. So, I didn’t go. In our village, especially for daughters-in-law, going out alone is not considered appropriate. Back in my maternal home, I used to accompany my sister-in-law for hospital visits, but here, the rules are different, and I am unfamiliar with the area. If someone from the family is available to go with me, then I step out. There is no strict restriction on going out, but I must have someone accompany me.”* |
|  | **Economic Barriers to care** | *“My husband is a daily wage laborer who earns daily wages, and if he takes a day off to take me to the hospital, we lose that day’s income. That’s why I avoided going unless there was a serious problem. Even when I wanted to go, we didn’t have enough money for transport, and private hospitals charged too much. The government hospital is free, but they don’t have all the tests, and we would still have to spend money on medicines and scans. So, we just waited and hoped everything would be fine.”*  *“When I was about to deliver, my family was very happy, but I lost my baby. In the hospital, I saw other families giving money to the nursing staff after childbirth because they had a healthy baby boy. I felt bad for myself. I don’t know why they were giving money, but only after receiving the money, the nurses’ hand over the babies. Sometimes, families give money out of happiness, but in some cases, the nurses themselves ask for more. I kept thinking if my baby had survived, would my family also have had to do the same?”*  *“I heard that some families give extra money to the ASHA worker, and she helps them more, she takes them to the hospital, and reminds them about check-ups. But we are poor; we cannot give anything. So, she doesn’t come to our house as often. I only went for check-ups when someone in my family was free to take me, but most of the time, I just stayed home. I thought I didn’t have any complications, so I thought it was okay not to visit the hospital regularly.”* |
|  | **Perceived and experienced quality of services in facilities** | *“When I gave birth to my second child (a daughter), I went to the government hospital in XX. They made me go through ten different rooms. We visited government hospital for an ultrasound, but no one even helped us there. They made us go through ten rooms, up-stairs and down. They took so much blood for blood test and then told us to come back on the three days to collect the report. After that, we didn’t go back. When no one was even willing to help us, why would we go again? Because of this experience, I didn’t visit the hospital during my recent pregnancy as well, not even once”*  *“The government hospital didn’t do anything. They just made me sit there. They kept saying that we should visit some other hospital. They told my family to take me wherever they wanted. We asked them to at least write something on the paper, but they didn’t provide anything. I had no hope of surviving… My brother and my entire family were terrified. Then, they took me out of the government hospital and took me to XX Hospital. We left from here (government hospital) at 11:30, and by midnight, we reached there (private hospital). At the government hospital, they said they would have to do surgery. My brother immediately took me out of there… If my brother hadn’t been there, I wouldn’t have survived. The government hospital didn’t even care for me at all…In private hospital I delivered my previous child normalyl”*  *“They told us to go to xx village, but when they discharged me, they didn’t provide an ambulance. We had to arrange one ourselves and pay for it. The nurses there were acting, as if, care depended on money, if you pay them, they take better care of you. If I gave money, they would look after me more; if someone else gave money, they would prioritize them. They were taking ₹2000-2500 for everything, and this was in a government hospital…They spoke with such arrogance. They even slapped me on my abdomen. When my sister argued with them for not taking care of me, they got up angrily from their sleep and hit me. I was already in so much pain, my womb had opened, and my baby had died inside me. Instead of helping, they threw everyone out of the room and left me alone…When they cleaned me, they did it roughly. They put their hands inside with so much force and pressed hard. It was painful. The way they handled me didn’t feel right. It felt like they were punishing me instead of helping. I still don’t understand why they treated me this way.”* |
| **Delay in Seeking Care** | **Perceived Need for Antenatal Care (ANC)** | *“I didn’t feel the need to go for check-ups because I had no issues. Pregnancy is natural, my mother-in-law always says that in our time, we never went to the hospital unless something was seriously wrong. So, I also thought it wasn’t necessary.”*  *“I had no problems in my last pregnancies, so I assumed this one would also be fine. That’s why I didn’t go for check-ups unless I felt unwell.”*  *“I had already told them in the beginning that I wouldn’t be able to go again and again. At that time, I had a goat and a young daughter to take care of, so I didn’t have enough time. That’s why I couldn’t go regularly, and my medicines were often delayed.”*  *“I wasn’t very eager to go. When I found out I was pregnant again, I didn’t tell anyone for a long time. If it turned out to be a girl, I knew there would be problems at home. So, I didn’t register my pregnancy early.”* |
|  | **Physical Accessibility of Appropriate Care** | *“We don’t have our vehicle and even if we arrange a private vehicle, the roads are so bad that it takes too long to reach the hospital. During the rainy season, it becomes even worse because of mud as my house is in between the fields. Sometimes, I avoid visiting the health facility because the road is full of mud and water.”* |
|  | **Dependence of the women for transportation** | *“I cannot go to the hospital on my own. Because I don’t know the way to the hospital, and I have never gone out alone. I can only go if my husband or with my mother-in-law. Once, I was in severe pain, but no one was at home to take me, so I had to endure it the whole night…My husband and mother-in-law decide where I should go and which doctor to see. I just have to follow them.”*  *“Once, I said I needed to go for a check-up in 5th or 6th month, but no one at home was available to take me. I couldn’t go alone. I asked the ASHA worker, but she said she couldn’t arrange a vehicle. So, I didn’t go. In our village, especially for daughters-in-law, going out alone is not considered appropriate. Back in my maternal home, I used to accompany my sister-in-law for hospital visits, but here, the rules are different, and I am unfamiliar with the area. If someone from the family is available to go with me, then I step out. There is no strict restriction on going out, but I must have someone accompany me.”*  *“We called the ambulances, but it took too long to arrive. By the time it reached, I had already delivered the baby at home…. And baby was not moving or crying. If the ambulance had come on time, maybe things would have been different.”*  *“The ambulance is big and doesn’t come inside our village because the roads are too narrow. We had to carry my sister-in-law in a cart to the main road so that she could get inside the ambulance. She was in so much pain.”* |
| **Inadequate Quality of Care Received at The Healthcare Facilities** | **Delays and Inappropriate Referrals** | *“During my labor pain, we first went to the village doctor, who checked the baby’s heartbeat using a machine. The heartbeat was fine at that time. But he refused to proceed and told us to get an ultrasound. So, we went to the government hospital (sub-district hospital), but they also refused to treat me, saying they needed an ultrasound first. They didn’t explain anything to me, just told my family that the baby’s heartbeat was getting weaker and that I needed to be taken somewhere else quickly. In government hospital (sub-district hospital), they didn’t do much. They just kept asking for an ultrasound and told us to leave because my condition was worsening. Later, they informed my husband that the baby had died in my womb, there was no heartbeat anymore. They asked us to go to other government hospital (district hospital) and get me admitted there. By 7–8 PM, we reached district hospital, where they completed some paperwork and checked me again. But instead of treating me, they told us to go to xx Hospital (Medical college). However, we didn’t go there. People believe that once someone is referred to xx hospital (Medical college), the chances of survival are very low. So, instead, my family decided to take me back home.”*  *“During my labor pain, I first went to the Government Hospital in xx, but they referred me to xx Hospital (Medical College) because my case was risky, my “Onaal” (placenta) was coming first. We were on our way there when my father and father-in-law, who were in xx, told us not to go. They said no one would be able to help us there since xx (medical college) was too far. We had already traveled quite a distance, but they insisted we come back and visit xx Hospital (government hospital other distict) in xy instead. We spoke to the vehicle driver, and he charged us ₹2,500 to take us there. In total, we spent around 4 to 5 hours traveling from one hospital to another. It still troubles me to think that if I had received care in xx hospital (sub-district hospital) itself, my baby might have survived, because during my check-up there, the heartbeat was still present. At xx hospital (hospital in another district), the doctor did a vaginal examination and checked my reports. They told me that my baby had already died inside my womb. They said they could not perform surgery there because the doctor responsible for C-sections was on leave. So, they referred me to xx hospital in xy (nearby state). But instead of going there, we went to a private hospital in xy."*  *“We had referred a patient from the CHC to the district hospital because her condition seemed a bit serious, and we did have the Gynaecologist, pediatrician and ventilators for the mother and new born. But her family immediately called a “Maulvi” and asked him what they should do. The “Maulvi” reassured them that everything would be fine here, and they just needed to stay there. He said something on call in the ear of a woman who was admitted for delivery. It looked like they were doing some “Kriya” or “Jhaad-phook”. The family refused to listen to us and kept saying that the Maulvi had advised them not to go. In another similar case, we had given a referral to a woman, but her family consulted a Maulvi and decided to stay. Later, her condition worsened, but by then, it was too late.”*  *~Staff nurse* |
|  | **Disorganized and Inappropriate Services at the Healthcare Facilities** | *“Going to the hospital is such a hassle. Every time, they ask for new documents, and we have to stand in long lines, just waiting. The whole day gets wasted running around. Once, I reached the hospital at 8:00 in the morning and had to stay there until after 2:00 in the afternoon just for a single blood test. The blood test happens in one building, the ultrasound somewhere else, and then the report, we don’t even get it the same day! We have to come back after a week just to collect it. Everything is scattered. One moment they send us here, the next moment somewhere else. There isn’t even a proper place to sit. In summer, we were forced to stand outside under the sun. It was getting exhausting, but what could have been done? No one listens to our struggles.”*  *“The ASHA worker comes, but she never explains anything to us. She just comes, writes in the card, and leaves. She does nothing for pregnant women. We don’t even know what check-ups we need or what precautions to take.”*  *“She only came once during my sixth month. She just noted down some details and left. She didn’t say anything about what I should be careful about or what I should do for my health.”* |
|  | **Inadequate Skill of Staff and Gaps in Clinical Management** | *“The doctors didn’t give me any medicine for high blood pressure (BP). They just said to check it regularly, and it would be fine. I had severe swelling all over my body. I also visited a private hospital, and they confirmed my BP was high. I asked for medicine, but they said it would harm me and that BP would go away on its own. They only gave me medicine for swelling, but my BP stayed high. Every time I had a headache, I got my BP checked, and it was always high, yet they still didn’t give me any treatment.”*  *“The doctors in govt. hospital did the ultrasound in such a hurry, how can I be sure they checked properly? They didn’t even tell me whether the umbilical cord was positioned up or down. When I later had a private ultrasound, the doctor mentioned that the cord was on the lower side, but the government hospital staff said nothing. If they had informed me about the complication, we would have opted for a C-section to save our baby.”*  *“I was taken to the government hospital, but no one checked me properly. I kept telling them that my ultrasound was scheduled for the next day and asked them to wait, but the nurse insisted they would do a check-up. The nurses were very young they first discussed with each other because no senior doctor was in the night, and later, without any proper examination, they just inserted their hands inside. At that moment, I felt an unbearable pain, so sudden and intense that I was completely overwhelmed with fear because soon after this examination the movement of my baby stopped. Later, a madam came, the one who does ultrasounds. She took me to the ultrasound room and checked me the same way, inserting her hand again. After that, she suddenly looked anxious, almost panicked. Then she said, the baby is no longer alive…I was shocked. I asked her why, what had happened. But she didn’t say anything, just remained silent. I looked at her and said, ‘If my baby didn’t die because of you, then why are you sweating? Why do you look so scared?’ But she had no answer. She just stood there quietly.”*  *“When I went into labor in the night, the nurse was asleep. No matter how much we called, she didn’t wake up. I delivered my baby alone, and for 30-40 minutes, no one came. The baby was just lying inside my clothes, and the placenta was still inside me… when my family members shouted too much then she came. But she spoke in arrogance and even slapped me on my stomach. When my sister argued, she got angry, hit me in the face, and asked everyone to go out of the room. I was in pain, my baby had died inside me, and they just left me alone. During cleaning, they were extremely rough. They put their hands inside with force and pressed my stomach hard. It was painful and felt more like punishment than care.”*  *“The doctors didn’t give me any medicine for high blood pressure (BP). They just said to check it regularly, and it would be fine. I had severe swelling all over my body. I also visited a private hospital, and they confirmed my BP was high. I asked for medicine, but they said it would harm me and that BP would go away on its own. They only gave me medicine for swelling, but my BP stayed high. Every time I had a headache, I got my BP checked, and it was always high, yet they still didn’t give me any treatment.”*  *“The doctors in govt. hospital did the ultrasound in such a hurry, how can I be sure they checked properly? They didn’t even tell me whether the umbilical cord was positioned up or down. When I later had a private ultrasound, the doctor mentioned that the cord was on the lower side, but the government hospital staff said nothing. If they had informed me about the complication, we would have opted for a C-section to save our baby.”* |
|  | **Infrastructural and Equipment Deficiencies** | *“The reality is, we don’t even have basic facilities here. There’s no baby warmer, no power backup, no ventilators, and, most importantly, no pediatrician or gynecologist. We try our best, but when things go wrong, the families hold us responsible. They say, ‘Because the government hospital lacks facilities, our baby died.’ But what can we do? We don’t have the resources to save such critical cases.”*  ~Staff Nurse  *“The doctor told me to get an ultrasound, but the hospital didn't have the machine. They asked me to go to another hospital far away. I had to go twice because they only do ultrasounds on certain days. It was very difficult for me to travel again and again in this condition. I was advised a Level II scan, but it was too expensive. We didn’t have enough money, so I couldn’t get it done. Later, the doctor said if we had known earlier, we could have done something for my baby, but we never got that chance.”* |
| **Lack of Postpartum Continuum and Bereavement Care** | **Lack of Post-Partum and Bereavement Care Protocols at The Health-System Level** | *“My chest had become completely hard, like a stone. The pain was unbearable, as if a heavy weight was pressing down on me. My sister-in-law had taken some medicine once, and her milk had dried up completely, even for her next child. She told me about it, but I was too afraid to take it. I got the medicine, but I never swallowed it. Instead, I waited for the milk to stop on its own. People have their own ways of dealing with these things. Some believe in remedies, like wearing an undershirt inside out. My mother-in-law told me to do it, so I did. I didn’t believe in it at first, but after a day or two, my milk started decreasing, and then it stopped completely. After that, I started thinking maybe it actually worked. At first, I doubted my mother-in-law’s words, but in the end, I felt like she was right.”*  *“When my milk started coming in, I used a machine to express it. But my mother and mother-in-law told me not to take out all the milk. They said to leave some in my breasts, or else if the baby’s soul lingered, the milk would keep coming. No one, no ASHA, no Anganwadi worker, ever told me anything about what to do. Since I wasn’t taking all the milk out, my chest felt heavy and painful for two or three days. But after that, it just dried up.”* |
|  | **Lack of Pressure on Health Systems for Documentation and Continuity of Care** |  |
|  | **Lack of Awareness of Need For Care and Entitlement Among Women with Stillbirth** | *“My supervisor madam gave me 45 days of leave, but I didn’t know whether it was official or not. Usually, maternity leave is for six months, but I was only given 45 days. My case was a bit complicated, so I needed more rest. That’s why I extended my leave for three more months, but all the leave after the 45 days was unpaid. I don’t even know if I got my full rights or not… but I had no choice but to take leave without pay”*  ~Government staff  *“My madam didn’t give me leave. I only got one week off, but she kept calling me again and again. She said, ‘Why do you need leave? Your baby didn’t survive, why do you need a whole month of rest?’ They thought that leave is only needed if the baby is alive. But my health was also suffering… I was weak, I was grieving. But no one understood that I needed rest too.”*  *~* House help woman |
| **Implications of Stillbirth on Women** | **Deep Grief and Constant Thoughts About the Loss** | *“I already have four daughters, and now, even this boy is gone… I do all the work, but my head starts hurting whenever I think about it. I keep wondering, if God had just given me a healthy baby, there would be no worries. But what can I do now? I am just exhausted.”*  *“I keep thinking that if my baby had survived, my family would have treated me better. But since there's no baby, I have no value to them. All of this is happening because I lost my child. If my baby were here, I would have mattered… but now, I don’t.”*  *“When you carry a baby for 9 or 10 months, your heart feels strange… how could it not? After keeping the baby in my womb for so long, when it was finally time to hold them in my arms, I had to bury them in the ground instead. That thought never leaves me. I lose my appetite, my head hurts… all I do is think about my baby. I keep thinking about the one I lost. If this baby had survived, I had planned to either get surgery or keep taking medicine… but now, after everything that happened, I don’t know what to do anymore.”*  *“When they came back after burying the baby, they told me the baby was in the machine, that because it was born early, it had been placed in an incubator. I only found out the truth after coming home… My sister told me later. They didn’t tell me earlier, fearing how I would react. I have already lost three children before this. The pain of losing a child is unbearable. I have given birth to three babies, and now I found out that this one is also gone...”*  *“All of this is happening because my baby is gone. If I had a child, I would be valued… But since I don’t, I don’t matter to anyone.”*  *“I’m always stressed… My head hurts a lot… I keep thinking about my baby. People say anything to me, and it adds to my tension. They taunt me, saying I killed my own baby… that I was the reason. These thoughts just keep running in my mind.”*  *“I feel like I went through so much suffering, yet I still lost my baby, and all my money was spent too… When I’m alone at home, I keep thinking… If my baby were here, I would be busy playing with them. I don’t say anything to anyone… I just call my mother.”*  *“What happened to my baby keeps me in constant tension… I keep worrying, will I ever have a child? If I do, will they survive? These thoughts keep coming to my mind every few days… This is happening to all the boys… And when it happens to boys, the pain feels even greater… Though both (boys and girls) are equal...”* |
|  | **Physical Health Issues** | *“When my milk started coming in, I used a machine to express it. But my mother and mother-in-law told me not to take out all the milk. They said to leave some in my breasts, or else if the baby’s soul lingered, the milk would keep coming. No one, no ASHA, no Anganwadi worker, ever told me anything about what to do. Since I wasn’t taking all the milk out, my chest felt heavy and painful for two or three days. But after that, it just dried up.”*  *“My chest had become completely hard, like a stone. The pain was unbearable, as if a heavy weight was pressing down on me. My sister-in-law had taken some medicine once, and her milk had dried up completely, even for her next child. She told me about it, but I was too afraid to take it. I got the medicine, but I never swallowed it. Instead, I waited for the milk to stop on its own. People have their own ways of dealing with these things. Some believe in remedies, like wearing an undershirt inside out. My mother-in-law told me to do it, so I did. I didn’t believe in it at first, but after a day or two, my milk started decreasing, and then it stopped completely. After that, I started thinking maybe it actually worked. At first, I doubted my mother-in-law’s words, but in the end, I felt like she was* right.” |
|  | **Reduced Family Care and Social Isolation** | *“Earlier, in-laws used to take care of everything and look after me properly. I didn’t have to do much work. But now, they put all the work on me. If I say I can’t do it, they just tell me, ‘How can you not? You have to work.”*  *“Earlier, when I used to greet people with 'Namaste' or 'Ram-Ram, (ways of greeting in India)' they would respond. But ever since I lost my last two babies, many women have stopped talking to me. This is why I don’t go out anymore, I don’t talk to anyone because no one talks to me. I have felt this change myself… Whenever I greet someone, they just ignore me. I’ve noticed this ever since I lost my second son. People say, ‘She kills her babies.’ That’s why I don’t speak to anyone anymore.”* |
|  | **Blame and Domestic Violence** | *“Everyone blames me. They keep saying, ‘It happened because of you… the baby had complications because of you.’ But I know I did nothing wrong. Still, ever since I lost my baby, they have made my life miserable.”*  *“They dragged me out of the house, holding my hand. At that moment, I was so furious, I didn’t know what I was saying… and then I left. All of this is happening because the baby is gone. If my baby had survived, I would have mattered… but now, without a child, I have no worth.”* |
|  | **Postpartum Practices** | *“The moment I took a bath, I felt such 'relief", as if that was all I needed. Then, I skipped a day and bathed again, and just like that, I was back in the kitchen, cooking for my children. No rest, no care, as if I had never just given birth. It was as if the struggles of a new mother didn’t matter. There was no postpartum rest for me, only endless work.”* |
