# Supplementary material for: Exploring pathways leading to stillbirths and gaps in postnatal care among affected women in a rural north Indian district: A qualitative study using the social autopsy lens
Source: PLoS One. 2026 May 13;21(5):e0347994. doi: 10.1371/journal.pone.0347994 (PMC13170853; doi:10.1371/journal.pone.0347994)
Supplement: S2 File — (DOCX) [file pone.0347994.s002.docx]

**TITLE:** Exploring pathways leading to stillbirths and gaps in postnatal care among affected women in a rural north Indian district: A qualitative study using the social autopsy lens

Barsha Gadapani Pathak^1,2^*, Sonia Maurya^1^, Shruti Bisht^1^, Pranay Vats^1^, Reema Mukerjee^3^, Vinod Kumar Anand^1^, Sarmila Mazumder^1^

1 Society for Applied Studies, New Delhi, India

2 Centre for Intervention Science in Maternal and Child Health, Centre for International Health, Department of Global Public Health and Primary Care, University of Bergen, Bergen, Norway

3 Division of Reproductive, Child Health and Nutrition, Indian Council of Medical Research, New Delhi, India

***Corresponding author:** Barsha Gadapani Pathak, University of Bergen, Bergen, Norway and Society for Applied Studies, New Delhi, India

Email: [Barsha.Pathak@student.uib.no](mailto:Barsha.Pathak@student.uib.no) and [barsha.pathak@sas.org.in](mailto:barsha.pathak@sas.org.in)

**S2 Box: Summarization of the implications of stillbirths on women**

| **S2 Box: Summarization of the implications of stillbirths on women** | |
| --- | --- |
| Deep Grief and Constant Thoughts About the Loss | *“I already have four daughters, and now, even this boy is gone… I do all the work, but my head starts hurting whenever I think about it. I keep wondering, if God had just given me a healthy baby, there would be no worries. But what can I do now? I am just exhausted.”*  *“I keep thinking that if my baby had survived, my family would have treated me better. But since there's no baby, I have no value to them. All of this is happening because I lost my child. If my baby were here, I would have mattered… but now, I don’t.”*  *“When you carry a baby for 9 or 10 months, your heart feels strange… how could it not? After keeping the baby in my womb for so long, when it was finally time to hold them in my arms, I had to bury them in the ground instead. That thought never leaves me. I lose my appetite, my head hurts… all I do is think about my baby. I keep thinking about the one I lost. If this baby had survived, I had planned to either get surgery or keep taking medicine… but now, after everything that happened, I don’t know what to do anymore.”*  *“When they came back after burying the baby, they told me the baby was in the machine, that because it was born early, it had been placed in an incubator. I only found out the truth after coming home… My sister told me later. They didn’t tell me earlier, fearing how I would react. I have already lost three children before this. The pain of losing a child is unbearable. I have given birth to three babies, and now I found out that this one is also gone...”*  *“All of this is happening because my baby is gone. If I had a child, I would be valued… But since I don’t, I don’t matter to anyone.”*  *“I’m always stressed… My head hurts a lot… I keep thinking about my baby. People say anything to me, and it adds to my tension. They taunt me, saying I killed my own baby… that I was the reason. These thoughts just keep running in my mind.”*  *“I feel like I went through so much suffering, yet I still lost my baby, and all my money was spent too… When I’m alone at home, I keep thinking… If my baby were here, I would be busy playing with them. I don’t say anything to anyone… I just call my mother.”*  *“What happened to my baby keeps me in constant tension… I keep worrying, will I ever have a child? If I do, will they survive? These thoughts keep coming to my mind every few days… This is happening to all the boys… And when it happens to boys, the pain feels even greater… Though both (boys and girls) are equal...”* |
| Physical Health Issues | *“When my milk started coming in, I used a machine to express it. But my mother and mother-in-law told me not to take out all the milk. They said to leave some in my breasts, or else if the baby’s soul lingered, the milk would keep coming. No one, no ASHA, no Anganwadi worker, ever told me anything about what to do. Since I wasn’t taking all the milk out, my chest felt heavy and painful for two or three days. But after that, it just dried up.”*  *“My chest had become completely hard, like a stone. The pain was unbearable, as if a heavy weight was pressing down on me. My sister-in-law had taken some medicine once, and her milk had dried up completely, even for her next child. She told me about it, but I was too afraid to take it. I got the medicine, but I never swallowed it. Instead, I waited for the milk to stop on its own. People have their own ways of dealing with these things. Some believe in remedies, like wearing an undershirt inside out. My mother-in-law told me to do it, so I did. I didn’t believe in it at first, but after a day or two, my milk started decreasing, and then it stopped completely. After that, I started thinking maybe it actually worked. At first, I doubted my mother-in-law’s words, but in the end, I felt like she was* right.” |
| Reduced Family Care and Social Isolation | *“Earlier, in-laws used to take care of everything and look after me properly. I didn’t have to do much work. But now, they put all the work on me. If I say I can’t do it, they just tell me, ‘How can you not? You have to work.”*  *“Earlier, when I used to greet people with 'Namaste' or 'Ram-Ram, (ways of greeting in India)' they would respond. But ever since I lost my last two babies, many women have stopped talking to me. This is why I don’t go out anymore, I don’t talk to anyone because no one talks to me. I have felt this change myself… Whenever I greet someone, they just ignore me. I’ve noticed this ever since I lost my second son. People say, ‘She kills her babies.’ That’s why I don’t speak to anyone anymore.”* |
| Blame and Domestic Violence | *“Everyone blames me. They keep saying, ‘It happened because of you… the baby had complications because of you.’ But I know I did nothing wrong. Still, ever since I lost my baby, they have made my life miserable.”*  *“They dragged me out of the house, holding my hand. At that moment, I was so furious, I didn’t know what I was saying… and then I left. All of this is happening because the baby is gone. If my baby had survived, I would have mattered… but now, without a child, I have no worth.”* |
| Postpartum Practices | *“The moment I took a bath, I felt such 'relief", as if that was all I needed. Then, I skipped a day and bathed again, and just like that, I was back in the kitchen, cooking for my children. No rest, no care, as if I had never just given birth. It was as if the struggles of a new mother didn’t matter. There was no postpartum rest for me, only endless work.”* |
